# Microneedles for Vaccination: Mechanistic Foundations, Materials Innovation, Clinical Translation, and Global Health Implementation

**DOI:** 10.3390/pharmaceutics18081022

**Published:** 2026-08-17

**Authors:** Hiep X. Nguyen, Mai Phuong Ho

**Affiliations:** College of Pharmacy, California Northstate University, 9700 West Taron Drive, Elk Grove, CA 95757, USA

**Keywords:** microneedle patch, intradermal vaccination, thermostability, decentralized manufacturing, immunization equity, combination-product regulation

## Abstract

Vaccination ranks among the most consequential interventions in medicine, yet cold-chain dependence, sharps hazards, needle phobia, and reliance on trained vaccinators limit coverage where vaccine-preventable mortality is highest. Microneedle patches deposit antigen into the antigen-presenting-cell-rich epidermis and upper dermis through projections that penetrate the stratum corneum without reaching dermal nociceptors. Such targeting yields immunogenicity matching or exceeding intramuscular injection at a fraction of the antigen mass, with dose-sparing up to six-fold recorded for influenza, polio, and SARS-CoV-2 antigens. Solid-state formulation converts that immunological advantage into a logistical one, since polymeric matrices preserve potency for as long as two years at ambient temperature, removing the refrigeration infrastructure that consumes significant delivery cost. Six microneedle types have reached preclinical or clinical maturity, with dissolving microneedle patches the most advanced. Two trials provide the clinical evidence: a Phase I influenza study showing non-inferior antibody responses and successful self-application, and a Phase I/II measles–rubella trial in The Gambia reaching 93% measles and 100% rubella seroconversion in infants without related serious adverse events. Engineering advances currently include an automated printer producing thermostable mRNA–lipid nanoparticle patches that retain bioactivity for six months at ambient temperature, the first intradermal self-amplifying RNA patch, and quantum-dot on-body immunization records. Sterility assurance, dose uniformity, nucleic acid integrity within solid matrices, and fragmented regulatory guidance remain the challenging obstacles, alongside a clinical pipeline concentrated on few antigens. This review integrates the mechanistic, materials, manufacturing, clinical, regulatory, and global health dimensions of the field to guide translation and equitable deployment.

## 1. Introduction

Vaccination averts an estimated 2 to 3 million deaths annually and remains among the most cost-effective preventive measures available, consuming only 2–3% of worldwide pharmaceutical spending [1]. Between 2011 and 2020, immunization programs across 94 low- and middle-income nations spent approximately $62 billion, of which delivery and supply-chain costs accounted for 54% and 6%, respectively [1]. Beyond individual protection, vaccines provide herd immunity that shields infants, pregnant women, older adults, and immunocompromised persons [2]. Coverage nonetheless remains uneven. Only 60% of eligible children in low- and middle-income regions receive adequate immunization [3], and 35% of sub-Saharan African children aged 12–23 months fail to complete the recommended schedule [4]. Non-adherence carries a measurable clinical cost across disease areas, exposing more than 40% of patients to significant health risks in some settings and contributing to an estimated 125,000 annual deaths in the United States [5,6]. Multi-dose schedules compound this risk: clinical programs for CV7201 (rabies), CV9103 (prostate cancer), and mRNA-4157-P201 (melanoma) require three, five, and nine administrations, respectively [7,8,9].

Hypodermic injection delivers approximately 3 billion of the more than 5 billion vaccine doses given each year [10], and this dominance carries substantial operational cost. Cold-chain maintenance consumes an estimated $200–300 million annually for refrigerated storage, shipment, and containers, a significant burden in tropical, remote, or politically unstable regions [1,11]. Between 25% and 50% of vaccines worldwide are wasted through cold-chain failure, with losses concentrated where vaccine-preventable mortality is highest [12,13,14]. Sharps injuries among healthcare workers range from 0.18 per professional per year in North America to 4.7 per provider annually in Egypt and Pakistan, generating roughly 3 million occupational injuries each year with attendant hepatitis B, hepatitis C, and human immunodeficiency virus risk [10]. Unsafe injection practices contribute to an estimated 1.3 million annual deaths [15,16]. Needle phobia affects at least 10% of the population and drives vaccine hesitancy, delayed boosters, and missed appointments [17]. Procedural anxiety, injection-site pain, hazardous biomedical waste, and unpredictable delivery depth further limit conventional administration [18,19,20].

Microneedles (MN) are micron-scale projections, typically 50–2000 µm tall and 1–100 µm wide at the base, arrayed on a polymeric or metallic backing [19,21]. Alza Corporation patented miniaturized needles in 1976, and Henry and colleagues demonstrated the first practical transdermal microneedle in 1998 [22], placing the field nearly three decades into engineering maturity rather than at conceptual inception. Microneedle insertion creates transient micron-scale channels through the stratum corneum, delivering antigens, nucleic acids, peptides, and small molecules into the epidermal and upper dermal compartments [23,24,25]. Insertion force ranges from 0.1 to 3 N, within the capability of thumb pressure or a spring-loaded applicator [26]. Pain perception falls by up to 90% relative to conventional subcutaneous needles [27], and skin recovers within approximately 100 min of array removal without lasting sequelae [28].

The clinical and public health value of microneedles originates from the combination of several attributes that address recognized deficiencies of hypodermic injection. Painless, self-applicable delivery reduces reliance on skilled vaccinators and eases the workforce strain of mass campaigns [22]. Solid-state formulation reduces or removes cold-chain requirements, with retention of vaccine activity at ambient and elevated temperatures for one month to two years depending on antigen and excipient system [29,30,31]. The absence of sharps waste removes needle-stick injury risk, simplifies disposal, and supports community-level and home-based administration [32,33,34]. Acceptability findings consistently favor patches over needle and syringe [35]. Recognizing these advantages, the World Health Organization, UNICEF, Gavi, PATH, and the Bill & Melinda Gates Foundation jointly prioritized microneedle array patches through the Vaccine Innovation Prioritization Strategy, while the Biomedical Advanced Research and Development Authority Patch Forward Prize reflects parallel United States investment in pandemic preparedness.

This review integrates immunology, materials chemistry, fabrication engineering, formulation science, clinical evidence, regulatory analysis, manufacturing scale-up, safety, global health implementation, and future research directions across the primary literature, consensus documents, and trial reports of the past decade, with an analytical focus that differs from other recent reviews. Berger and colleagues [36] delivered the first meta-analysis of immunogenicity, safety, and acceptability data across microarray patch trials but were limited to influenza and Japanese encephalitis antigens and preceded the pediatric measles–rubella dataset that has since anchored the field. Zheng and coworkers [22] surveyed microneedle biomedical devices as a device class across drug delivery and biosensing, treating vaccination as one use case among several. Nguyen and Nguyen [25] reviewed microneedle-mediated biopharmaceutical delivery across therapeutic modalities. Against this background, the present review contributes five analytical elements. First, we integrate cross-trial immunogenicity and thermostability data, including the Adigweme 2024 Gambian pediatric measles–rubella dataset [37] published after the Berger search cutoff, with the mechanistic detail linking the observed dose-sparing to skin-resident antigen-presenting cell density and lymphatic trafficking kinetics, and reinterpret the Berger heterogeneity finding as clinical and methodological rather than biological in origin. Second, we perform a biomechanically grounded failure-mode analysis of the Prins negative outcome, reading the trial as an empirical test of the murine-to-human translational gap rather than as a case of geometric mismatch alone. Third, we position every principal vaccine target and platform combination on a two-dimensional map of clinical evidence versus manufacturing readiness, with readiness gated by four sequential requirements (dose uniformity, sterility assurance, Good Manufacturing Practice scale-up, and harmonized regulatory acceptance), exposing a pattern that individual trial reports cannot reveal. Fourth, we consolidate the sterility, isolator, and process analytical technology questions that determine whether a laboratory-stage patch can reach licensure, with quantitative data on radiation and thermal effects on nucleic acids, lipid nanoparticles, protein antigens, and each principal polymer class, and connect these to the convergence with mRNA design, machine learning for structure-activity optimization, and wearable biosensing that will shape the coming decade. Fifth, we frame the five- and ten-year research direction as a structured roadmap of falsifiable milestones, with equitable deployment in low- and middle-income settings as the yardstick against which those milestones are set.

## 2. Immunological and Pharmacological Rationale for Skin-Targeted Vaccination

### 2.1. The Stratum Corneum Barrier and Its Mechanical Circumvention

The stratum corneum measures 10–30 µm in adult human skin and consists of 15–20 layers of anucleate corneocytes embedded in a lamellar matrix of ceramides, free fatty acids, and cholesterol [38]. This brick-and-mortar composite excludes molecules above approximately 500 Da, charged species, and hydrophilic macromolecules [39], restricting passive diffusion to small lipophilic compounds and rendering the intact barrier unsuitable for vaccine antigens [19,25,40]. Microneedles bypass the barrier mechanically rather than chemically, generating aqueous channels that connect the skin surface to the viable epidermis and upper dermis [25,41]. These channels remain short enough to avoid the dermal nociceptor network, which accounts for the marked pain reduction observed clinically [41]. Channel longevity is finite, with closure typically complete within two hours of array removal [42,43], and barrier function recovers without detectable long-term compromise [44,45].

### 2.2. Skin-Resident Antigen-Presenting Cells and Lymphatic Trafficking

**Figure 1:**
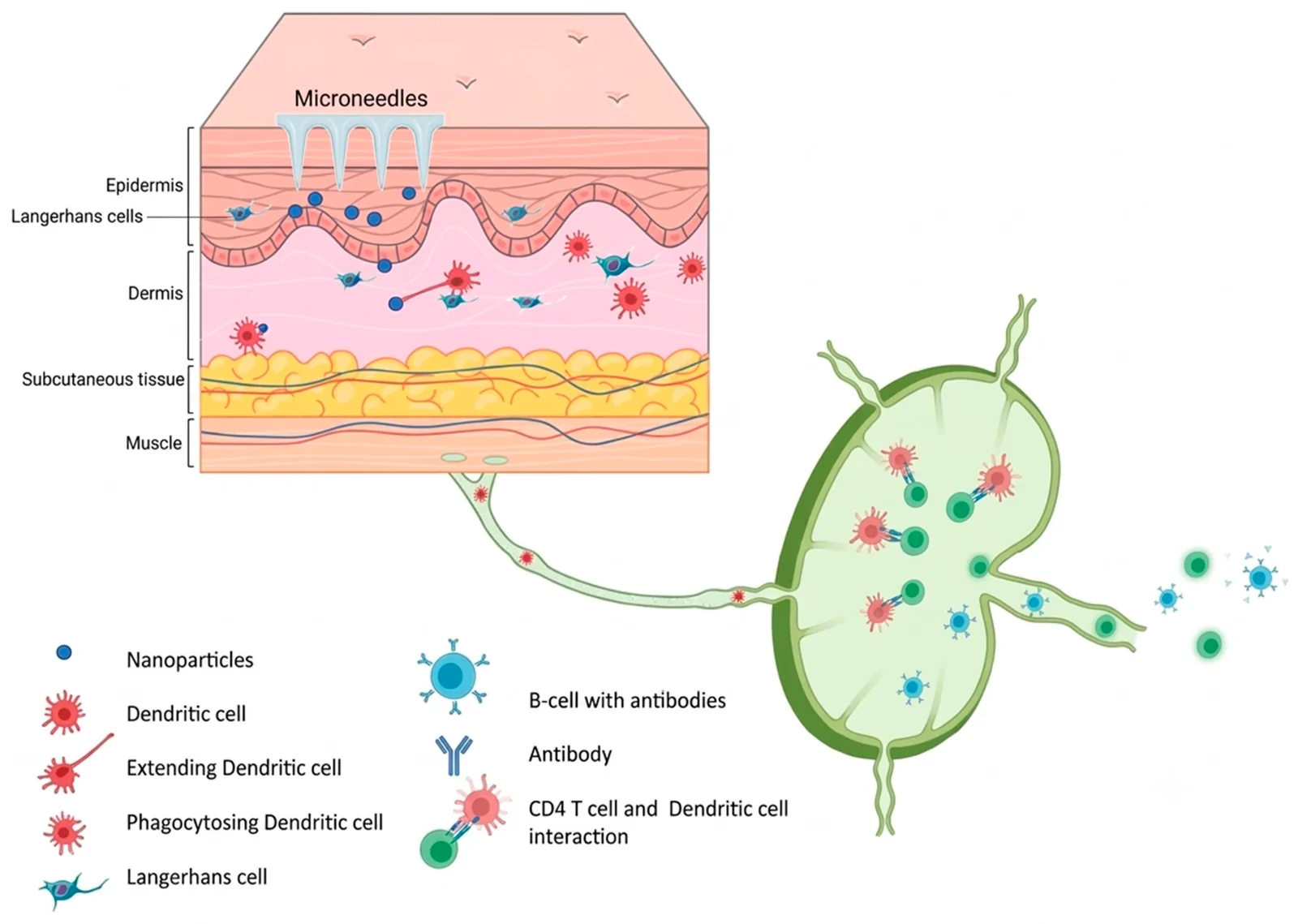
Skin structure and the cellular immunology underpinning microneedle-based vaccination. The skin comprises the avascular epidermis, the vascularized dermis, and subcutaneous adipose tissue, separated from muscle by connective tissue. Microneedle insertion through the stratum corneum delivers antigen directly into the epidermal and upper dermal compartments. Langerhans cells resident in the epidermis and dermal dendritic cells in the dermis capture and process the antigen, traffic to skin-draining lymph nodes through the lymphatic vasculature, and engage CD4^+^ T cells and B cells to drive germinal-center formation and antigen-specific antibody production. Images reprinted with permission from [50].

Skin functions as both a physical barrier and an immunologically active interface. The tissue comprises the avascular epidermis, the vascularized dermis of roughly 1–4 mm, and subcutaneous adipose tissue, and houses T lymphocytes, epidermal antigen-presenting cells, dermal macrophages, and skin-associated lymphoid tissue [38,46,47]. Langerhans cells, identified by CD1a and langerin (CD207) expression, populate the epidermis at 500–1000 cells per mm^2^ and capture antigen through constitutive endocytosis [48]. The upper dermis contains roughly ten-fold greater dendritic cell density per unit volume than circulating blood [49], and this numerical advantage supplies the cellular basis for clinical dose-sparing. Figure 1 illustrates the skin structure and cellular immunology underpinning microneedle-based vaccination.

Upon antigen capture, Langerhans cells downregulate adhesion molecules, mobilize from the epidermis, and migrate through dermal lymphatics to draining lymph nodes, retaining antigen for 2–3 days during transit and thereby sustaining presentation [51]. Within the lymph node paracortex, both populations present processed peptides on MHC class I and II complexes to naive CD8^+^ and CD4^+^ T cells and supply the costimulatory and cytokine signals required for clonal expansion [52]. Insertion itself generates a localized sterile inflammatory microenvironment characterized by monocyte-derived dendritic cell recruitment, keratinocyte cytokine release, and upregulated pattern recognition receptor expression [53], functioning as an endogenous adjuvant signal.

Poor dermal vascularization, frequently regarded as a delivery constraint, provides an immunological benefit: antigen deposited in the epidermis and upper dermis persists locally longer than material injected into highly vascularized muscle, increasing the probability of capture by resident dendritic cells [54]. Microneedle-delivered vaccines generate significantly more germinal center B cells than matched intradermal bolus injection [55], and sustained presentation over 7–28 days improves memory B cell frequency and CD8^+^ T cell recall through iterative cycles of somatic hypermutation. A Phase I immunological substudy of the inactivated influenza dissolving patch reported higher proportions of circulating CD4^+^CXCR5^+^CXCR3^+^ICOS^+^PD-1^+^ T follicular helper cells in patch recipients than in intramuscular controls on day 8, alongside superior neuraminidase inhibition titers across all three vaccine strains [56].

Pharmacokinetic data support the immunological rationale for skin-targeted delivery. Table 1 summarizes the reported comparisons across delivery routes. Fluorescence tracking after coated-microneedle influenza delivery in mice showed antigen persistence at the skin insertion site for at least 3 days, with matured antigen-loaded dendritic cells detectable in draining lymph nodes at 24 h, whereas intramuscularly administered antigen cleared substantially faster from the deposition site [57]. Reddy and colleagues quantified size-dependent lymphatic transport after intradermal administration: 25 nm PEG-stabilized nanoparticles drained efficiently through lymphatic capillaries and were taken up by approximately half of lymph-node-resident dendritic cells, whereas 100 nm particles were largely retained at the injection site, revealing that particulate cargo below 50 nm partitions preferentially into lymphatic clearance rather than local retention [58].

Deposition depth within the 100–1100 µm range determines the response through the layered anatomy of skin-resident antigen-presenting cell populations. Liard and coworkers compared subcutaneous, intradermal, and transcutaneous routes for HIV-1 p24 particulate antigen and observed route-specific differences: subcutaneous delivery yielded p24-specific IgG without detectable antigen-specific CD8^+^ T cells; intradermal delivery generated both humoral and cellular responses; and transcutaneous application through hair follicles, which preferentially engages epidermal Langerhans cells, elicited CD8^+^ effector responses with mucosal IgA and without systemic IgG [59]. This pattern is consistent with the depth distribution of resident cells (epidermal Langerhans cells at 50–100 µm; papillary dermal dendritic cells at 300–700 µm; reticular dermis and blood-vessel network below 700 µm) [49,60]. Quantitative depth-stratified partitioning of lymphatic versus systemic clearance across the microneedle operating range has not been reported in humans and represents an important priority for future translational pharmacokinetic studies.

### 2.3. Dose-Sparing Relative to Intramuscular Delivery

Dose-sparing is the most reproducible immunological advantage of microneedle vaccination and appears consistently across influenza, polio, SARS-CoV-2, hepatitis B, and Japanese encephalitis antigens. Lower intradermal doses generate immunity equal to or exceeding equivalent intramuscular preparations, with protection at one-quarter to one-tenth of conventional dosing [61]. Delivery of inactivated influenza vaccine using a high-density microarray patch (MAP) achieved non-inferior hemagglutination inhibition titers at one-sixth the intramuscular antigen mass [62]. Polio vaccination by microneedle application reached equivalent antibody titers using one-quarter of the conventional dose, thereby addressing the supply constraints of inactivated poliovirus vaccine production [63]. Table 2 consolidates validated dose-sparing effects of microneedle-based vaccination.

The dose-sparing effect translates into vaccine supply economics. During pandemic surges, when manufacturing capacity becomes rate-limiting, achieving protection with one-sixth to one-tenth of the conventional antigen mass would expand global supply substantially [62]. For antigens whose production yields cannot readily scale, such as inactivated poliovirus, dose-sparing converts into expanded coverage [63]. Dose-sparing nonetheless remains insufficiently characterized in regulatory submissions, because most published trials are powered for immunogenicity equivalence at matched doses rather than for the formal dose–response comparisons required to support a fractional-dose label claim. Future Phase II and III programs should incorporate dose-finding cohorts that quantify the achievable magnitude in humans.

### 2.4. Mechanobiology of Insertion

Successful insertion requires applied force exceeding the elastic and viscoelastic resistance of skin while remaining below the threshold for needle fracture. Davis and colleagues demonstrated a linear relationship between insertion force and needle tip cross-sectional area, with measured forces of 0.1–3 N suitable for manual application [26]. Needle length is constrained by competing factors: microneedles must bypass the stratum corneum and reach the antigen-presenting-cell-rich compartments, yet remain short enough to spare deeper nociceptors [55]. Penetration depths between approximately 100 and 1100 µm define the operational window for painless, immunologically effective delivery [67].

Skin biomechanics introduce substantial inter-individual variability. Skin thickness differs by anatomical site, age, body mass index, ethnicity, and hydration state [19], and therefore a fixed-length array reaches different absolute depths across individuals. Standardized spring-loaded or latch-based applicators constitute an essential element of product design rather than an optional accessory.

## 3. Microneedle Classes and Design Principles

**Figure 2:**
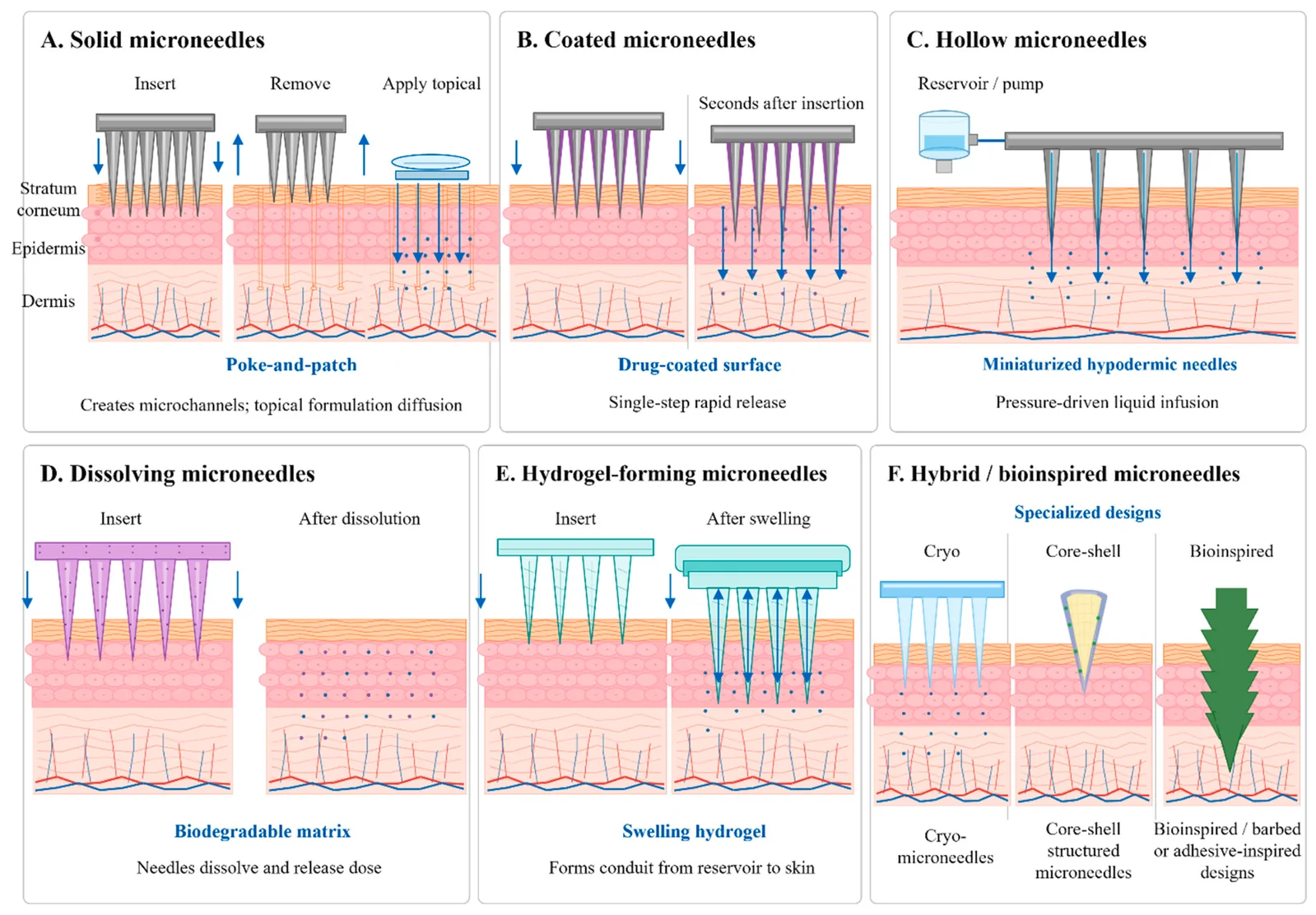
The six microneedle types for transcutaneous vaccine delivery. (**A**) Solid microneedles operate through a poke-and-patch sequence, in which the array first generates transient microchannels before topical application of the vaccine formulation. (**B**) Coated microneedles use the coat-and-poke principle, with vaccine deposited as a dry film on the needle surface and released upon insertion. (**C**) Hollow microneedles deliver liquid formulations through internal lumens connected to a reservoir. (**D**) Dissolving microneedles use the poke-and-release principle, in which the polymeric or sugar matrix containing the cargo dissolves entirely within the skin. (**E**) Hydrogel-forming microneedles release cargo by swelling-mediated diffusion from an attached reservoir, with the intact matrix removed cleanly after application. (**F**) Hybrid and bioinspired microneedles, including cryo-microneedles, core–shell-structured microneedles, and bioinspired/barbed or adhesive-inspired designs.

Two decades of design optimization have produced six microneedle families distinguished by cargo deposition mechanism, materials, fabrication route, and release kinetics. Figure 2 summarizes the six microneedle types together with their mechanisms.

Solid microneedles originated in the late 1990s with silicon arrays applied to transdermal delivery [68]. The poke-and-patch sequence inserts the array to create microchannels, removes the array, and then applies a topical formulation that diffuses through the disrupted barrier [41,53]. Silicon, stainless steel, titanium, and polymers serve as substrates [69,70,71]. Solid arrays have also functioned as electrodes concentrating the electric field within the epidermis during DNA vaccine electroporation, improving transfection relative to intramuscular electroporation [72,73]. Rapid microchannel closure, a cumbersome two-step sequence, residual surface drug, and non-degradable waste nonetheless constrain broad vaccine use [53].

Coated microneedles deposit the formulation as a thin film on solid projections before insertion [74]. The aqueous interstitial environment dissolves the coating within seconds, releasing cargo in a single step. Kim and colleagues coated stainless steel needles with methylcellulose or hydroxyethyl cellulose plus trehalose, preserving hemagglutinin activity through three months at room temperature and providing complete protection against lethal influenza challenge [75]. Vrdoljak and coworkers achieved the first intradermal inoculation of recombinant live viral vectors by coating arrays with adenovirus and modified vaccinia virus Ankara [76]. Loading below roughly 10 µg per needle suits potent antigens such as influenza hemagglutinin but restricts application to vaccines requiring milligram doses [77].

Hollow microneedles function as miniaturized hypodermic needles, delivering liquid formulations under pressure-driven flow through internal lumens [43,78,79]. The format accepts conventional liquid formulations without solid-state development, leaves no polymer residue, and accommodates larger volumes than coated or dissolving microneedles [80,81]. The MicronJet600 device (NanoPass Technologies) is the most clinically advanced hollow microneedle system, having supported human trials against influenza, rabies, hepatitis B, polio, and BCG [82]. Reduced mechanical strength, lumen clogging, interfacial leakage, and greater reported pain limit wider adoption [83,84].

Dissolving microneedles are the most clinically advanced vaccine platform. Biodegradable, water-soluble polymers and sugars embed the antigen within the needle matrix, which dissolves in interstitial fluid within minutes and leaves no sharp residue [41,70,80,85]. Common matrices include polyvinylpyrrolidone, polyvinyl alcohol, carboxymethylcellulose, hyaluronic acid, poly(lactic-co-glycolic acid), trehalose, sucrose, and maltodextrin [41,80]. The poke-and-release mechanism deposits the full dose quantitatively at the intradermal site regardless of microchannel resealing or coating adherence, a mechanistic advantage over both passive diffusion and surface desorption. Two pivotal trials confirmed clinical viability: Rouphael and colleagues demonstrated safety, tolerability, non-inferior immunogenicity, and successful self-application for inactivated trivalent influenza vaccine in 100 healthy adults [33,34], and Adigweme and colleagues delivered measles–rubella vaccine to infants, toddlers, and adults in The Gambia with high seroconversion and a favorable safety profile [37].

Hydrogel-forming microneedles consist of cross-linked hydrophilic networks that absorb interstitial fluid and swell to form transient conduits through which a reservoir payload diffuses [41,86]. The matrix remains intact and is removed cleanly after use [87], and reservoir placement at the array base permits larger volumes than tip-loaded dissolving needles [81]. Courtenay and coworkers paired Gantrez S-97 arrays with lyophilized ovalbumin reservoirs and obtained IgG responses comparable to dissolving needles while permitting complete needle withdrawal [88]. Unintended reservoir leakage, inconsistent swelling across skin types, and modest mechanical strength have constrained commercialization [89,90].

Hybrid and bioinspired designs address specific limitations. Cryo-microneedles are frozen biologic-laden formulations molded into needle shapes that preserve live cells and mRNA vaccines, although sub-zero storage until application limits practical deployment [41,91]. Core–shell configurations house cargo in an inner core beneath a stabilizing shell; trehalose and sucrose formulations preserved recombinant SARS-CoV-2 S1-RBD antigen for one hour at 100 °C and four months at 37 °C [92,93].

Across all classes, three geometric parameters govern the balance among penetration efficiency, payload, and comfort: length, aspect ratio, and array density. High aspect ratios penetrate readily but fracture easily; low aspect ratios resist fracture but require greater insertion force. Density above 2000 needles per patch increases total surface area and dose capacity yet introduces manufacturing tolerance problems and may concentrate local trauma [94]; using several hundred to approximately 1500 needles per patch typically balances these factors. Table 3 compares the six classes to support microneedle type selection.

## 4. Materials Science

Material selection reconciles a tightly linked set of requirements: mechanical strength sufficient to penetrate the stratum corneum without fracture, biocompatibility consistent with intradermal deposition, compatibility with the cargo during fabrication and storage, dissolution kinetics matched to the intended release profile, and sustainability across manufacture and disposal. No single material satisfies every requirement, and practice consists of trade-offs among inorganic substrates, natural biopolymers, synthetic polymers, sugar matrices, and stimuli-responsive systems.

### 4.1. Inorganic Substrates

Silicon dominated first-generation development, offering tensile strength of roughly 1000–7000 MPa and a Young’s modulus of 130–188 GPa [27,42,95,96]. Brittleness, low payload capacity, high cost, multistep cleanroom processing, and concern over fragments retained in the dermis after fracture have curtailed clinical use [42,97].

Stainless steel combines 580 MPa tensile strength with a 193 GPa modulus, wide availability, scalability, biocompatibility, and low cost, making the alloy the most widely adopted inorganic alternative [43,70,98,99], although rigidity, non-biodegradability, corrosion, and nickel-related allergic risk remain concerns. Titanium offers 240–550 MPa tensile strength and biocompatibility with minimal corrosion at higher material cost [42,100,101]. Among ceramics, alumina is biocompatible but brittle and non-degradable, while calcium sulfate and calcium phosphate variants degrade but require multistep manufacture [42,98,102,103]. The nanoporous ceramic alumina array evaluated in the proof-of-concept SARS-CoV-2 trial by Prins and colleagues belongs to this class [20].

### 4.2. Natural Biopolymers

Hyaluronic acid, a non-sulfated glycosaminoglycan native to the dermis, has become one of the most widely used dissolving matrices. The polymer engages CD44, LYVE1, and RHAMM receptors on dermal dendritic cells and lymphatic endothelium, enabling receptor-mediated uptake [104,105]. Mechanical strength is modest, with a Young’s modulus near 39.9 ± 6.7 kPa when cross-linked with 10% PEG 2000, and payload capacity is correspondingly limited [98,106].

Chitosan, obtained by deacetylation of crustacean chitin, combines biocompatibility, biodegradability, intrinsic antimicrobial activity, wound-healing capacity, and generally-recognized-as-safe status. The polycationic charge forms favorable electrostatic contacts with keratinocyte and dendritic cell membranes, promoting uptake of co-loaded antigen. Implantable chitosan arrays carrying influenza vaccine elicited significantly higher virus-specific IgG than intramuscular delivery and afforded complete survival against H1N1 challenge, compared with 60% survival after intramuscular immunization, reflecting both depot behavior sustaining release over 28 days and the adjuvant properties of chitosan, including NLRP3 inflammasome activation [107]. Poor solubility requires multistep processing [108,109].

Silk fibroin from *Bombyx mori* stabilizes cargo through β-sheet crystalline domains that form nanopores immobilizing biological payloads and reducing water content and molecular mobility [110,111]. Silk films preserved inactivated polio vaccine at room temperature for three years with 70% potency retention and maintained roughly 50% potency across serotypes after 12 months at 45 °C [112].

### 4.3. Synthetic Polymers and Sugar Matrices

Synthetic polymers offer greater batch consistency, processability, and tunable degradation than natural biopolymers, and currently dominate clinical-stage dissolving microneedle arrays. Poly(lactic-co-glycolic acid) hydrolyzes into lactic and glycolic acid, both natural metabolic intermediates, with degradation tunable through monomer ratio and molecular weight [113]. Nanoparticles encapsulating ovalbumin and the TLR3 agonist poly(I:C), delivered through hollow arrays, generated balanced humoral and cellular responses [114]. Polyvinylpyrrolidone offers strong water solubility, biocompatibility, and regulatory acceptance, dissolving within minutes and suiting rapid-burst release [115,116]. Systematic screening for mRNA–lipid nanoparticle compatibility identified polyvinyl alcohol at mass fractions above 50% as the most effective colloidal stabilizer, with binary polyvinylpyrrolidone–polyvinyl-alcohol blends resolving the slow drying, hygroscopicity, and high viscosity of alcohol-only formulations while preserving nanoparticle size, ultrastructure, and mRNA integrity after reconstitution [117].

Sugar matrices serve dual structural and stabilizing roles. Trehalose, sucrose, maltose, raffinose, mannitol, sorbitol, maltodextrin, dextran, and inulin appear most frequently, often with arginine, histidine, calcium heptagluconate, or sodium citrate as supplementary stabilizers [118,119]. Stabilization proceeds through two complementary mechanisms. Vitrification forms an amorphous glassy matrix that immobilizes cargo, restricting the molecular motion that drives protein unfolding and aggregation [120,121]. Water replacement forms hydrogen bonds between sugar molecules and the hydration shells of proteins or membrane lipids, substituting for water removed during drying without compromising structure [122]. Trehalose protection extends across measles, rubella, and influenza antigens, with concentrations near 1% delivering marked benefits [123,124].

### 4.4. Stimuli-Responsive Materials, Biocompatibility, and Sustainability

Stimuli-responsive systems release cargo on demand in response to internal stimuli or external triggers. External triggers include insertion pressure, near-infrared and ultraviolet light, electric current, and temperature; internal triggers include glucose, hypoxia, reactive oxygen species, pH, thrombin, and bacterial enzymes [41]. pH-responsive systems use the mildly acidic microenvironment of inflamed skin to trigger antigen release [125,126], and enzyme-responsive designs degrade in response to matrix metalloproteinases for tumor-proximal delivery [125,127].

As the intradermal route places materials in direct contact with immunologically active tissue, biocompatibility carries particular weight. Polyvinylpyrrolidone, polyvinyl alcohol, poly(lactic-co-glycolic acid), hyaluronic acid, chitosan, and silk fibroin all carry favorable profiles supported by extensive use in approved medical products. Sustainability is gaining prominence as the field anticipates large-scale deployment. Biodegradable and dissolvable systems offer a path toward zero-waste administration [128], and cellulose-based materials support single-use device manufacture without the persistent waste generated by silicon, ceramic, or non-degradable polymer alternatives [129]. Eliminating sharps waste further removes the requirement for the medical waste incineration infrastructure that currently consumes substantial energy [77,130]. Table 4 presents the principal material classes.

## 5. Fabrication and Decentralized Manufacturing

Translation from laboratory prototype to licensed product depends on scalable, reproducible, Good-Manufacturing-Practice-compliant fabrication. Each method imposes constraints on geometry, polymer compatibility, cargo stability, throughput, and unit cost, and the chosen route shapes downstream regulatory, scalability, and commercial outcomes.

### 5.1. Micromolding, Lithography, and Laser Machining

Micromolding with polydimethylsiloxane molds remains dominant for dissolving arrays and accounts for most clinical-stage products. A viscous polymer-antigen solution is cast into a precision-machined mold, drawn into the cavities by centrifugation, vacuum, or pressure, and dried under controlled humidity and temperature [137]. Two-step casting has become the preferred technique for concentrating antigen at the needle tips, where dermal penetration is most efficient, while minimizing waste in the backing. Scaling to industrial volumes nonetheless presents engineering difficulties: polydimethylsiloxane molds tolerate neither high temperature nor high pressure, show limited dimensional stability across thermal gradients, and absorb small hydrophobic molecules from the casting solution [138]. Lot-to-lot variation in polymer molecular weight, viscosity, and moisture uptake represents a principal manufacturing concern under Good Manufacturing Practice conditions [138,139,140].

Photolithography and laser micromachining, borrowed from microelectronics, produced the earliest silicon and metal arrays. Photolithography resolves sub-100 µm features but is multistep, labor-intensive, expensive, and limited to simple uniform geometries [77,141]. Both photolithography and wire drawing require processing temperatures near 100 °C, which reduces vaccine integrity and confines these routes to solid or coated needle types in which payload is deposited after fabrication [142]. Laser ablation and three-dimensional laser cutting afford precise control over geometry and permit customization by pairing computer-aided design with high-energy beams that shape stainless steel or titanium sheets [77,143,144]. Centrifugal and draw lithography draw viscous polymer solutions upward into needle structures at ambient temperature, preserving thermolabile cargo, and underpin commercial microneedle production in Korea [132,145].

### 5.2. Three-Dimensional Printing

Three-dimensional printing offers rapid prototyping, geometric customization, and potential point-of-care manufacture. Stereolithography photopolymerizes liquid resin layer by layer under an ultraviolet laser, and insulin-coated arrays produced by this method achieved steady glucose control relative to subcutaneous injection [146,147,148,149]. The photocurable chemistry of most resins nonetheless yields densely cross-linked, water-insoluble matrices [150], precluding encapsulation within the needle body and restricting the method to coating-based loading. Digital light processing cures each layer with a projector rather than a scanning laser, increasing throughput while retaining the same material constraint [151,152], and suits master mold production and prototyping [151].

Continuous liquid interface production directs ultraviolet light through an oxygen-permeable window beneath the resin bath, curing layers continuously rather than stepwise and yielding rapid print rates with smooth surfaces [153,154]. Caudill and coworkers used this method to produce 700 µm polyethylene glycol needles bearing horizontal grooves that expanded loading surface area, achieving 36% higher antigen loading than square pyramidal geometries and inducing a Th1-biased response to ovalbumin plus CpG through prolonged skin retention [153]. Inkjet printing deposits precise micrometer-scale droplets of vaccine and polymer ink under ambient conditions without thermal or radiation exposure.

### 5.3. Decentralized Vaccine Printers

The microneedle vaccine printer reported by Vander Straeten and colleagues integrates an automated robotic dispenser, programmable vacuum chamber, and modular motion stages into a standalone device capable of fabricating thermostable mRNA patches at the point of care [117]. The system addresses the central logistical obstacle to mRNA deployment in low- and middle-income countries, where ultra-cold-chain dependence and limited trained personnel translate into constrained access. Three principal operations define the workflow: loading, dispensing, and drying. Vacuum-based filling uses the permeability and solubility of air in polydimethylsiloxane to draw viscous inks into the cavities without bubble formation, providing a repeatable, automatable process that scales to any patch size or quantity [155]. The first-generation device produces 100 patches in 48 h, with throughput scalable through modular drying rack expansion, parallel dispensing lines, and robotic patch retrieval [117].

Ink formulation followed systematic screening of dissolvable polymers for compatibility with lipid nanoparticles approximately 147 nm in diameter encapsulating mRNA [156]. Quality outputs include correctly formed sharp needles in 100% of tip-loaded patches and consistent DNA loading across a 100-mold tray with a standard deviation of 1.6 µg and no positional trend. Encapsulated mRNA loading reaches approximately 1.0 µg per patch with low batch-to-batch variation, and patches retain bioactivity for at least six months at 4 °C or 25 °C and one month at 37 °C [117]. Simultaneous tip-loading of multiple cargoes falls within the device capability [117].

The microfluidic microneedle array patch described by Driskill and colleagues offers a complementary route. Designed in Autodesk Fusion and fabricated by injection continuous liquid interface production, the device integrates a microfluidic channel network with an internal reservoir storing lyophilized vaccine [157]. These platforms show that mRNA and self-amplifying RNA patches can in principle be produced with compact automated equipment, offering a potentially more resilient model for pandemic response than the centralized, cold-chain-dependent system exposed as vulnerable during the COVID-19 pandemic. The current evidence derives from prototype-scale devices operated under research conditions. Neither the microneedle vaccine printer nor the microfluidic array patch has been validated under current Good Manufacturing Practice, and neither has quantified the delivered dose reaching the epidermis and upper dermis in human volunteers. Reported quality metrics such as tip-formation success rate, intra-tray content uniformity, and in vitro bioactivity retention document reproducible fabrication under laboratory control but do not substitute for aseptic-process validation, sterility assurance at the parenteral standard of 10^−6^, or dye-tracer confirmation of the fraction of loaded cargo reaching the target compartment. Closing these gaps is discussed as a principal engineering priority in Section 10, and the platforms are characterized as promising rather than implementation-ready [117,158,159]. Table 5 compares the principal methods for microneedle fabrication.

## 6. Vaccine Cargo Engineering and Thermostabilization

Product performance depends as much on the cargo formulation embedded within the matrix as on needle geometry and materials. Formulation must reconcile two competing requirements: preservation of antigen integrity through the mechanical, thermal, and chemical stresses of fabrication, storage, and in-skin dissolution; and presentation of the antigen in a form that elicits protective immunity upon intradermal deposition.

### 6.1. Protein, Inactivated, Live-Attenuated, and Particulate Antigens

Subunit vaccines composed of purified proteins, glycoproteins, peptides, and recombinant antigens adapt most readily to microneedle formulation because purified preparations are stable, compatible with polymer embedding, and free of the biosafety concerns attached to live pathogens. Lower intrinsic immunogenicity typically requires adjuvant co-incorporation, and recombinant proteins degrade substantially under prolonged ambient exposure [61,165], thus manufacture requires stabilizing excipients and tightly regulated drying. Nanoparticle carrier selection shapes the qualitative character of the response: liposomes outperformed poly(lactic-co-glycolic acid) and other carriers for CD4^+^ and CD8^+^ T cell induction [114], reflecting superior membrane fusogenicity and endosomal escape that support MHC class I cross-presentation.

Inactivated vaccines are generally more thermostable than live-attenuated preparations [166], and inactivated antigens delivered by microneedle retained stability for three weeks at 50 °C, whereas conventional preparations lost 40% of antigenic content within seven days [167]. Live-attenuated formulations pose a harder problem because viability must survive drying, storage, and application, and because reversion risk and the narrow +2 °C to +8 °C thermal window constrain acceptable matrices and processes [122]. Even so, live-attenuated vaccines delivered by microneedle have shown stronger immunogenicity than subcutaneous injection alongside better heat tolerance, retaining 90% of potency after four months at 40 °C [168].

Virus-like particles reproduce the outer structure of viruses and preserve neutralizing epitopes without carrying a viral genome [169], combining whole-virus immunogenicity with subunit safety. The Nanopatch system delivered human papillomavirus vaccine in this format without exogenous adjuvant, relying on the immunostimulatory properties of intradermal delivery itself [35].

### 6.2. Nucleic Acid Vaccines

DNA vaccines drive antigen expression from plasmid vectors, with subsequent MHC class I and II presentation activating CD4^+^ and CD8^+^ T cells [52,170]. Molecular stability permits long-term ambient storage and large-scale bacterial fermentation, providing a substantial cost advantage over in vitro transcription [53,171]. Wijesundara and colleagues delivered dry-loaded pVAX-tpaNS1 Zika DNA through a high-density microarray patch that remained stable for 28 days at 40 °C and induced stronger mucosal and systemic anti-NS1 IgG and T cell responses than conventional intradermal vaccination [172].

Messenger RNA offers short development timelines, scalability, high efficiency, and rapid sequence optimization [53]. Stability remains the central formulation problem: mRNA is intrinsically labile and susceptible to ubiquitous RNases and requires cryogenic storage under conventional liquid conditions [117]. Lipid nanoparticles shield the payload and promote intracellular uptake, yet nanoparticle stability at elevated temperatures constrains potency and worldwide availability [92]. Both mRNA-1273 and BNT162b2 use N1-methyl-pseudouridine with a modified 5′ cap [173,174,175], achieving over 90% protection in trials, whereas the unmodified-nucleoside candidate CVnCoV reached only 48% efficacy in the HERALD Phase IIb/III trial [176]. Koh and coworkers showed that naked mRNA dissolved in polyvinylpyrrolidone can transfect skin cells and induce cellular and humoral responses [177], suggesting that the disrupted epithelial barrier may partially substitute for nanoparticle-mediated endosomal escape; if extensible to clinically relevant antigens, this finding would remove the most costly component of mRNA manufacture.

Self-amplifying RNA encodes an alphavirus-derived replication complex that replicates intracellularly, generating large quantities of antigen-encoding transcript from a single delivered molecule [178]. Driskill and colleagues reported the first intradermal delivery of self-amplifying RNA by microarray patch, achieving antibody and T cell responses comparable to intradermal and intramuscular controls from either liquid or lyophilized payloads, with immunogenicity preserved for at least 15 weeks under warm, non-freezing storage [159]. Lyoprotectant optimization has been conducted and found effective: at 5 and 10% (*w*/*v*) sucrose, insufficient glass-matrix formation permitted vesicle fusion during freezing, yielding post-reconstitution diameters of 225 and 192 nm with hexagonal aggregation, whereas 20% sucrose preserved spatial separation and yielded 135 nm particles within the range favoring antigen-presenting cell uptake [159,179].

### 6.3. Adjuvant Co-Formulation

Adjuvants supply the pattern recognition receptor activation required alongside antigen for full antigen-presenting cell licensing, and microneedles place both components in defined spatial proximity within the same needle, maximizing co-uptake by individual cells [55]. CpG oligodeoxynucleotides co-delivered with ovalbumin produced Th1-biased humoral and cellular responses [153]. Poly(I:C) co-encapsulated with ovalbumin in poly(lactic-co-glycolic acid) nanoparticles generated balanced responses [114]. Shifting the ratio of co-formulated adjuvants altered antigen-presenting cell phenotypes [180], showing that combinatorial design permits fine control over innate signaling. For nucleic acid cargoes, the mRNA molecule itself carries pathogen-associated molecular patterns that engage TLR3, TLR7, TLR8, RIG-I, and MDA5, providing self-adjuvancy that pseudouridine substitution deliberately attenuates to improve translation [181], a trade-off directly relevant to formulation decisions. Localized cellular necrosis triggered by insertion also serves as an endogenous immunostimulant [182], permitting adjuvant-free formulations such as the Nanopatch human papillomavirus vaccine [183].

### 6.4. Thermostabilization and Cold-Chain-Free Formulation

Thermostabilization is arguably the most consequential pharmaceutical objective in the field, bearing directly on vaccine access, supply-chain economics, and pandemic preparedness. Dissolving microneedles provide enhanced stability through three complementary mechanisms: the polymer matrix restricts molecular mobility and prevents aggregation, low water activity suppresses hydrolytic degradation, and the absence of freeze–thaw cycling removes a major source of protein and nanoparticle damage [93,184]. Solid-state formulations consequently outperform liquid preparations, remove the need for reconstitution, and lessen cold-chain reliance [153,185,186].

The principal stabilization chemistries fall into four categories: physical protection through encapsulation and matrix embedding (silk fibroin, poly(lactic-co-glycolic acid), polyethylene glycol hydrogels); water removal by lyophilization, vaporization, foam drying, spray drying, or electrospray; chemical excipient stabilization through vitrification and water replacement; and protein or genetic engineering of antigens and carriers for intrinsic stability. Performance across these strategies is notable. Optimized binary and ternary combinations of trehalose, sucrose, arginine, and calcium heptagluconate maintained most or all inactivated influenza immunogenic activity over 24 months at 25 °C [119]. A pentavalent formulation combining diphtheria, tetanus, pertussis, hepatitis B, and *Haemophilus influenzae* type b antigens retained complete antigenicity across 12 months at 25 °C [30].

Physicochemical characterization has clarified the mechanisms by which solid matrices preserve lipid nanoparticle integrity. Three interdependent parameters control the shelf life: the glass transition temperature (Tg) of the dried matrix, the residual moisture content following secondary drying, and the preservation of the ionizable-lipid/mRNA core enveloped by helper phospholipids that defines the intact bilayer organization [187]. Differential scanning calorimetry confirms that trehalose forms glasses with Tg near 115–120 °C in the dry amorphous state, sucrose near 65–75 °C, and glucose considerably lower, thus trehalose provides a wider thermal margin against plasticization under tropical storage conditions [188]. X-ray diffraction of well-formulated preparations shows the broad amorphous halo characteristic of vitrified sugar matrices rather than the sharp Bragg peaks that would signal recrystallization and consequent loss of water-replacement hydrogen bonding at the lipid headgroup interface. Residual moisture also requires careful control: AboulFotouh and coworkers reported that RNA–LNP dry powders with moisture below approximately 0.5% or above 3–3.5% *w*/*w* showed increased particle size and reduced encapsulation efficiency, defining an operating window in which residual water plasticizes without driving hydrolysis [189]. Cryogenic electron microscopy of reconstituted lyophilized mRNA–LNPs preserves the electron-dense core–shell morphology of the parent dispersion, with mRNA and ionizable lipid confined to the aqueous core and phospholipids forming the outer boundary [190,191]. In the polyvinylpyrrolidone–polyvinyl-alcohol matrix of the vaccine printer, polyvinyl alcohol hydroxyls serve as water surrogates that hydrogen-bond with lipid headgroups and stabilize the outer shell, whereas polyvinylpyrrolidone contributes matrix rigidity and rapid drying kinetics that shorten the exposure of LNPs to aqueous stress during solidification; the combined system limits lateral lipid diffusion and suppresses the fusion and phase-separation events that would otherwise drive irreversible aggregation [117]. Dynamic light scattering confirms that reconstituted LNPs from optimized dried matrices show only a moderate diameter increase relative to fresh dispersions, and encapsulation efficiency correlates directly with size preservation, since particle coalescence during drying releases mRNA into the surrounding matrix where the payload becomes susceptible to hydrolytic scission; formulations that hold the mean diameter within approximately 20% of the initial value retain encapsulation efficiency above 90% and in vivo translation indistinguishable from the controls [190,192]. These parameters define a rational specification for critical quality attributes of dried mRNA–LNP microneedle products and provide the mechanistic basis for the thermostability outcomes tabulated in Table 6.

Interpretation of thermostability data requires attention to what the retention assay measures. Antigenicity refers to the ability of an antibody to recognize the antigen and is documented by binding assays such as enzyme-linked immunosorbent assay, single radial immunodiffusion, Western blot, or receptor-binding assays [193]. Biological activity refers to the functional properties on which the vaccine depends and is documented by infectivity assays for live-attenuated and viral-vector vaccines (TCID_50_, focus-forming unit, plaque assay), by hemagglutination inhibition for influenza, by in vitro transfection or translation reporters for nucleic-acid vaccines, and by enzyme-linked immunosorbent assays targeting conformational epitopes such as the D-antigen for inactivated poliovirus, whose signal correlates with functional protection [193]. Protective efficacy refers to demonstration that the stored preparation protects against pathogen challenge in an animal model or meets a clinically accepted correlate of protection in humans, and is not implied by antigenicity or biological activity alone. A vaccine that retains binding epitopes can lose infectivity, and a preparation that retains in vitro function can still fail to protect in vivo, so the three categories are neither redundant nor interchangeable.

Table 6 summarizes thermostability performance across antigen classes. Retention metric and assay method differ by cargo type: enzyme-linked immunosorbent assay (ELISA) and single radial immunodiffusion (SRID) for protein subunit and inactivated antigens, plaque or focus-forming unit assays and TCID_50_ for live-attenuated preparations, in vitro transfection or translation measurements together with RiboGreen encapsulation for nucleic acids, and in vivo immunogenicity as the common functional measure across classes.

**Table 6:** Thermostability performance for representative microneedle vaccine systems.

Antigen Class	Formulation	Storage Condition	Retention	Study Level	Evaluation Method ^a^	References
Recombinant subunit (HBsAg)	Dissolving MN, adjuvant-free	20–25 °C, 6 months	67 ± 6% of initial potency	Preclinical, mice and rhesus macaques	In vivo anti-HBs IgG ELISA in mice and rhesus macaques	[194]
Live-attenuated (measles–rubella)	Dissolving MN, sucrose/threonine/CMC	40 °C, ≥1 month; ambient long-term	≥90% viral titer retained	Preclinical, rhesus macaques	TCID_50_ in Vero cells; in vivo neutralizing antibody	[124,168]
Live-attenuated (rotavirus)	Lyophilized, sugar-based	45 °C, 7 months	≥85% infectivity retained	In vitro	Focus-forming unit (FFU) assay	[195]
Live-attenuated viral vectors (adenovirus, MVA poxvirus)	Carbohydrate glass film	45 °C, 6 months	≤0.5 log_10_ titer loss (~68%)	In vitro	TCID_50_ in HeLa cells	[196]
Inactivated pentavalent (DTP–HepB–Hib)	Dissolving MN	25 °C, 12 months	Antigenicity indistinguishable from control for all five components	Preclinical, mice	Antigen-specific ELISA per component	[30]
Inactivated influenza (coated)	Sucrose or trehalose	25 °C, 12 months	HA content unchanged; antibody response indistinguishable from fresh	Preclinical, mice	SRID for HA content; HAI and in vivo antibody titers	[29]
Inactivated influenza (multi-excipient sugar glass)	Trehalose/sucrose/arginine/calcium heptagluconate	25 °C, 24 months	Most or all HA activity retained	Preclinical, mice	SRID; HAI; in vivo immunogenicity	[119]
Inactivated poliovirus	Silk fibroin film	Room temperature, 3 years; 45 °C, 12 months	70% D-antigen at RT/3 y; ~50% at 45 °C/12 mo	In vitro	Type-specific D-antigen ELISA	[112]
DNA (SARS-CoV-2 S and N)	Chitosan oligosaccharide MN	Room temperature, >1 month	Neutralizing activity indistinguishable from freshly prepared control	Preclinical, mice	Pseudovirus neutralization; in vivo IgG ELISA	[197]
DNA (Zika NS1)	HD-MAP, dry-loaded	40 °C, 28 days	DNA integrity preserved; anti-NS1 IgG and T cell responses maintained	Preclinical, mice	Agarose gel electrophoresis; in vivo IgG ELISA and IFN-γ ELISpot	[172]
Recombinant SARS-CoV-2 S1-RBD	Core–shell trehalose/sucrose MN	100 °C, 1 h; 37 °C, 4 months	RBD binding preserved; in vivo IgG comparable to fresh control	Preclinical, rats	RBD–hACE2 binding ELISA; in vivo IgG ELISA	[93]
mRNA–LNP	PVP–PVA MN via vaccine printer	4 °C and 25 °C, 6 months; 37 °C, 1 month	Bioactivity retained without measurable loss under the reported conditions	In vitro and preclinical, mice	In vitro firefly luciferase transfection; in vivo IgG ELISA	[117]
mRNA–LNP	Tray lyophilization, sugar-based	25 °C, 12 weeks; 4 °C, 24 weeks	<10% change in mRNA integrity and encapsulation efficiency	In vitro and preclinical, mice	RiboGreen encapsulation efficiency; capillary gel electrophoresis for mRNA integrity; in vitro transfection	[190,192]
saRNA–LNP (lyophilized)	iCLIP M-MAP	25 °C, 15 weeks	Immunogenicity indistinguishable from fresh liquid control	Preclinical, mice	In vitro translation reporter; in vivo IgG ELISA and pseudovirus neutralization	[159]

^a^ Each row is categorized by the highest level of retention evidence reported in the primary source: antigenicity (structural or epitope preservation by binding assay), biological activity (infectivity, translation, hemagglutination inhibition, or receptor-binding function), or protective efficacy (in vivo immunogenicity meeting a seroprotection correlate, or challenge protection in an animal model).

Protective efficacy after thermostabilized storage has been demonstrated in only a subset of the systems in Table 6. The clearest preclinical evidence comes from Joyce and colleagues, whose study showed that measles vaccine microneedle patches induced neutralizing antibodies in rhesus macaques at titers exceeding the accepted seroprotection threshold and protected against measles virus challenge after storage under the reported conditions [168]. Alcock and colleagues showed that adenovirus and modified vaccinia Ankara vectors stabilized on the carbohydrate glass film retained protective immunogenicity in mice after six months at 45 °C [196]. Multiple influenza microneedle patches have documented lethal H1N1 challenge protection in mice with survival rates approaching 100% after storage at ambient or elevated temperature. Clinical-level correlates of protection have been met in the Rouphael Phase I trial, in which hemagglutination inhibition titers ≥1:40 (the accepted correlate for influenza) were reached after intradermal dissolving microneedle delivery [34]; in the Forster HD-MAP dose-sparing study, in which non-inferior hemagglutination inhibition titers were reached at one-sixth the intramuscular dose [62]; and in the Adigweme Phase I/II measles-rubella trial in The Gambia, in which 93% measles and 100% rubella seroconversion was recorded in infants receiving the microarray patch, meeting the World Health Organization seroprotection correlates for both antigens [37]. For nucleic-acid systems, protective efficacy after thermostable storage is limited to preclinical species, with the Vander Straeten mRNA vaccine printer and the Driskill saRNA microfluidic patch reporting IgG and neutralizing responses in mice after storage but no human challenge data [117,159]. The asymmetry between physical stability data and challenge-level efficacy data is one of the field’s most critical evidentiary gaps and is addressed in the roadmap discussion in Section 12.

Computational and machine-learning methods for rational design of heat-stable variants form a developing area that requires thorough experimental confirmation before practical use [151]. The translational implications are considerable. Ambient storage and distribution remove the most expensive and infrastructure-intensive component of vaccine logistics. In Australia, where cold-chain logistics consume 7–10% of total influenza vaccine cost, cost-benefit modeling projected $99 million in savings and sharply reduced wastage from adopting thermostable microneedle immunization [198]. Economic advantages were found strongest for high-demand, wide-coverage vaccines, including measles, yellow fever, and influenza [199].

## 7. Clinical Evidence Against Infectious Diseases

Several published clinical trials cover influenza, measles, rubella, SARS-CoV-2, polio, hepatitis B, rabies, tuberculosis, dengue, and Zika virus.

### 7.1. Influenza

Rouphael and colleagues randomized 100 healthy adults aged 18–49 to a health-worker-applied dissolving patch, a self-applied patch, intramuscular injection, or placebo patch [34]. Both patch arms produced antibody responses non-inferior to injection at matched dose, 70% of participants preferred the patch at 28 days, self-administration succeeded without prior training, and the product remained thermostable outside the cold chain [34]. The accompanying immunological substudy found higher neuraminidase inhibition titers across all three strains and a greater percentage of circulating T follicular helper cells on day 8 relative to intramuscular controls [56], consistent with antigen deposition in a compartment dense with professional antigen-presenting cells and favorably drained by lymphatics. The same substudy reported higher IL-5 and IL-13 concentrations in patch recipients, consistent with short-term local reactogenicity attributable to epidermal disruption [56].

Dose-sparing is the most reproducible finding across influenza trials. Forster and colleagues showed that a 2.5 µg high-density microarray patch dose achieved immunogenicity statistically indistinguishable from 15 µg intramuscular, a six-fold antigen saving [62]. The Vaxess MIMIX dissolvable patch has advanced to Phase I evaluation for H1N1 influenza (NCT06125717) [200].

### 7.2. Measles and Rubella

The Adigweme Phase I/II trial in The Gambia represents the most consequential clinical milestone achieved to date and the strongest evidence base for pediatric vaccination in a low- and middle-income setting [37]. The study recruited 45 adults, 120 toddlers aged 15–18 months, and 120 infants aged 9–10 months under a double-blind, double-dummy, randomized, active-controlled design, with age de-escalation from adults to toddlers to infants and independent Data Monitoring Committee review of 14-day safety data between cohorts [37].

Among measles-seronegative infants aged 9–10 months, the principal audience for measles vaccination in endemic regions, 93% seroconverted after patch delivery versus 90% after subcutaneous injection, while both routes reached 100% rubella seroconversion [37]. These rates met or exceeded the historically reported 85–90% following subcutaneous vaccination at 9 months. At day 180, measles seroprotection reached 91% for the patch and 93% for subcutaneous delivery, with 100% of infants in both groups remaining rubella-seropositive. Geometric mean concentrations of measles-neutralizing antibodies continued rising from day 42 to day 180 in both arms, a pattern indicating continued viral replication and antigen synthesis consistent with a primary response in this cohort [37]. In previously vaccinated toddlers, measles geometric mean concentration rose from 572.8 to 2182.9 mIU/mL with the patch versus 566.9 to 1811.5 mIU/mL with subcutaneous delivery, and in adults from 242.7 to 1107.4 mIU/mL versus 199.2 to 590.2 mIU/mL [37]. Joyce and colleagues had previously shown that the same technology protected infant rhesus macaques completely against measles challenge [201]. The product has advanced to Phase III evaluation, and the Bill & Melinda Gates Foundation funded the trial through the collaboration between Micron Biomedical and the Medical Research Council Unit, The Gambia [202].

### 7.3. SARS-CoV-2 and Other Targets

The COVID-19 pandemic exposed the vulnerability of conventional delivery to dependence on trained personnel, centralized cold chains, and needle-and-syringe throughput. Intracutaneous delivery of SARS-CoV-2 subunit sequences through an electroporation-enabled array induced protection in rats equivalent to intramuscular inoculation at a ten-fold higher dose [65].

The proof-of-concept trial by Prins and collaborators provides the field’s most analytically informative negative result [20]. Delivery of 20 µg mRNA-1273 through nanoporous ceramic alumina arrays failed to induce an anamnestic antibody response, whereas intramuscular administration of the identical dose produced a strong anamnestic booster response. The authors attributed the failure specifically to the vaccine loading technique, which produced insufficient tip-localized dose despite an average delivery efficiency near 68% [20]. The mechanistic reading benefits from a fuller consideration of the biomechanical mismatch between the murine or ex vivo human tissue used in preclinical development and the living human skin encountered in the clinic.

Human skin behaves as a viscoelastic composite with strain-rate-dependent stiffness and site-, age-, and hydration-sensitive mechanical properties. In vivo indentation measurements report an effective Young’s modulus of 10–100 kPa for the near-surface dermis, increasing into the megapascal range in the reticular dermis under tensile loading, while the dry stratum corneum measures 100–1000 MPa and softens by roughly an order of magnitude upon full hydration [203,204]. Under a spring-loaded applicator, the loading rate falls within the range where stress relaxation and viscous drag reduce the effective stiffness felt by the needle tip, yet the elastic recoil that follows initial deformation drives the skin surface back toward the applicator plane and can reduce the fraction of needle length actually embedded to 30–70% of the nominal value [26,205]. For a nanoporous ceramic array whose payload release depends on interstitial fluid ingress into micron-scale channels, incomplete embedding shortens the diffusion path and reduces the fluid volume available to solubilize the mRNA–lipid nanoparticle cargo, effects that contribute to the release shortfall cited by Prins and colleagues.

Stratum corneum thickness compounds the difference. Human stratum corneum measures 10–30 µm across most body sites and reaches 200–400 µm on palmoplantar surfaces [206], while murine dorsal stratum corneum is typically 5–15 µm and rests on a dermis of only 0.5–1 mm supported by the panniculus carnosus, a subcutaneous striated muscle absent from most human body sites [207,208]. The panniculus supplies both a compliant substrate that eases insertion and a mechanical stop that limits insertion-depth variability in murine studies, neither of which operates in human trials. Porcine skin, with a stratum corneum of 15–25 µm and a dermis of 1–3 mm, is a closer analog and should be the preferred late-preclinical model for any array intended for human intradermal delivery [207]. The consequence is that a geometry validated in murine skin typically over-penetrates relative to intent, whereas a geometry validated in ex vivo human skin, which lacks in vivo blood pressure, interstitial fluid turnover, and living viscoelastic response, typically under-performs in the clinic.

Interstitial fluid pressure adds a further layer that is often overlooked. Dermal interstitial pressure falls between −2 and 0 mmHg under baseline conditions and increases modestly with local edema, and the transient microtrauma created by array insertion generates a brief hyperemic and edematous response that increases local fluid availability over the first minutes of wear [209]. For dissolving matrices, this fluid influx is sufficient to complete matrix dissolution within the wear period. For a non-dissolving nanoporous ceramic, whose payload must diffuse through fixed channels of a few tens to hundreds of nanometers, the rate of fluid uptake, the wetting angle of the porous alumina surface, and the desorption kinetics of adsorbed mRNA–lipid nanoparticles control how much of the loaded dose reaches the skin. A mismatch between nominal loading (measured by content-uniformity assay before application) and delivered dose (measured by residual assay after removal) has been repeatedly documented for the ceramic platform class [20,210]. Combined with the loss of embedded needle length attributable to viscoelastic recoil, the release kinetics observed in ex vivo Franz-cell studies or in murine skin cannot be reliably extrapolated to the human clinical setting.

The Prins outcome therefore reads less as a case of poor geometry and more as a confluence of three mismatched assumptions: that murine and ex vivo human tissue predict the viscoelastic response of living human skin; that nominal patch loading equates to delivered dose in a non-dissolving porous scaffold; and that iterative geometric optimization can compensate for a material choice whose release chemistry is tied to variables the patch does not control. The result carries a broader translational message. Geometry and release optimization for any non-dissolving intradermal platform require early human volunteer studies with dye-tracer or radiolabel confirmation of delivered dose, rather than preclinical extrapolation alone, and the Section 12 discussion of insertion-depth variability [19,22,151] should be read as an empirical corollary of this trial. Porcine ex vivo and in vivo bridging studies, standardized applicators that constrain loading rate within the window where skin viscosity dominates the response, and delivered-dose acceptance criteria that treat nominal loading and released fraction as separate critical quality attributes offer the most direct path toward avoiding a repeat of this outcome.

For inactivated poliovirus vaccine, rhesus macaque studies yielded neutralizing concentrations equivalent to intramuscular vaccination for serotypes 1 and 2 [211], and dose-sparing at one-quarter of conventional antigen [63] aligns directly with production constraints faced by the Global Polio Eradication Initiative. Fractional inactivated poliovirus delivered through the MicronJet600 device has been recommended by the WHO Strategic Advisory Group of Experts as a cost-reduction measure [212]. For hepatitis B, Choi and colleagues delivered adjuvant-free surface antigen through a dissolving patch that induced innate and adaptive responses comparable to conventional vaccines and retained 67 ± 6% potency after six months at 20–25 °C [194]. This figure requires careful contextualization: licensed hepatitis B vaccines are expected to retain potency within the label-claim specification throughout the assigned shelf life, and therefore a 33% loss within six months would not satisfy prequalification criteria, illustrating the gap between academic proof-of-concept and regulatory-grade stability [53]. Rabies DNA vaccination in Beagle dogs by minimally trained staff matched intramuscular immunogenicity [53,213], an operationally consequential result for canine-transmitted rabies programs.

Among emerging and neglected pathogens, a first-in-human trial of PepGNP-Dengue delivered a gold nanoparticle multivalent peptide candidate through 600 µm solid silicon needles, although the absence of a non-microneedle comparator arm limits attribution of benefit to the route [151,214]. Skin and mucosal surfaces share an integrated immunological network mediated by lymphocyte trafficking, common cytokine pathways, and overlapping dendritic cell populations, hence intradermal vaccination can generate mucosal responses through skin–mucosa cross-talk; a recombinant HIV-1 CN54gp140 microneedle prime followed by mucosal boost illustrates this axis [215], and transcutaneous delivery of cationic nanoparticle–DNA complexes induced strong mucosal immunity with balanced Th1/Th2 polarization [216]. Table 7 presents the principal clinical trials.

## 8. Cancer Vaccines and Immunotherapy

Cancer vaccination by microneedle uses the dense antigen-presenting cell network of skin to prime tumor-specific T cells outside the immunosuppressive tumor milieu, supports co-delivery of antigen with diverse immunomodulatory cargoes, and permits localized deposition that limits the systemic toxicity of checkpoint blockade. An interesting proof-of-concept illustrates the potency of skin-targeted immunostimulation: array delivery of murine melanoma cells induced tumors ten-fold larger than those produced by 125-fold more cells delivered through a hypodermic needle [218], indicating significantly more efficient cellular trafficking and microenvironment formation.

Personalized neoantigen vaccination represents a promising application. Co-delivery of neoantigens with Toll-like receptor agonists through polyelectrolyte multilayered coatings offers a generalizable platform [219], and prophylactic tumor-antigen vaccination by microneedle generates systemic memory T effector cells [55,220]. Since clinical personalized schedules require five to nine administrations, the painless, self-applicable, thermostable patch format could substantially improve adherence and thereby the viability of mRNA-based cancer immunotherapy [7,8,9]. Photothermal and photodynamic strategies combine light-activated cytotoxicity with immune stimulation, ablating tumor tissue while exposing antigen for in situ priming [221], with synergistic efficacy when paired with checkpoint blockade [222,223,224].

**Figure 3:**
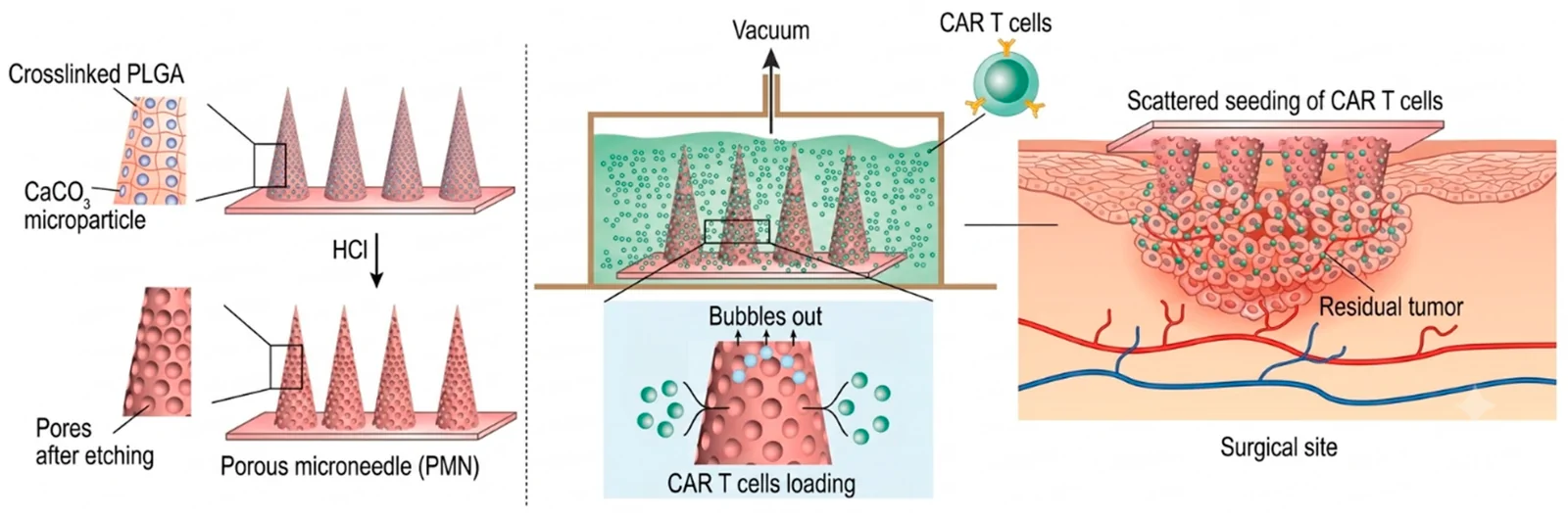
Engineered porous microneedles for chimeric antigen receptor T cell delivery to the surgical tumor bed. Cross-linked poly(lactic-co-glycolic acid) microneedles embedded with calcium carbonate microparticles undergo hydrochloric acid-mediated etching to generate an interconnected porous structure. CAR T cells are subsequently loaded into the porous matrix under vacuum, with simultaneous extraction of trapped air bubbles ensuring uniform cellular distribution within the needle. Application of the loaded patch to the surgical resection cavity enables scattered seeding of CAR T cells across the residual tumor bed, providing localized cellular immunotherapy targeted at minimal residual disease. Images reprinted with permission from [227].

Checkpoint inhibitor co-delivery directs agents specifically to tumor tissue or skin-draining lymph nodes, improving CD8^+^ T cell infiltration and promoting inflammatory polarization of tumor-infiltrating immune cells [224,225]. PD-L1 siRNA combined with systemic anti-PD-1 antibody achieved 83.3% long-term survival in murine melanoma [73], and STAT3 siRNA delivery produced up to 80% tumor volume reduction at 264 µg with a dose-dependent profile that validates the precision dosing capacity of dissolving arrays. Prostate cancer DNA vaccination by microneedle generated tumor-specific cellular immunity against a deep-seated tumor [226], and tumor-antigen DNA delivered to the backs of mice prevented internal lung metastases [136], confirming that skin-targeted priming can extend protection to distant sites. A porous platform for chimeric antigen receptor T cell delivery to the surgical tumor bed illustrates a further application, summarized in Figure 3.

Two constraints have been reported and discussed. First, for tumors not located in or beneath the skin, direct intradermal targeting is anatomically mismatched, and therapeutic reach depends on circulating tumor-specific T cells generated by skin priming [53]. Second, the small volume of dissolving needles limits absolute dose; for milligram-scale monoclonal antibody therapeutics, loading capacity is categorically insufficient without substantial increases in patch area, and delivering infusion-scale immunotherapy doses may not be volumetrically feasible [55]. Microneedle-based oncology applications therefore concentrate on antigens, adjuvants, nucleic acids, and small molecules deliverable at low dose with high efficiency.

## 9. Innovations

Five advances define the current development beyond first-generation dissolving microneedle patches.

**Decentralized thermostable mRNA manufacture.** The microneedle vaccine printer offers a proof-of-concept for self-contained modular fabrication that reduces the labor-intensive manual steps of conventional dissolving-patch casting [228]. Bioactivity retention of at least 6 months at 4 °C and 25 °C and 1 month at 37 °C, documented in vitro and in mice, would in principle satisfy the logistical requirements for ambient distribution across tropical and remote settings [117]. A network of such printers producing patches on site from centrally designed digital sequences would offer a potentially more resilient pandemic response than centralized cold-chain-dependent supply, contingent on cGMP-compliant aseptic processing, dose-uniformity acceptance criteria compatible with USP <905>, and delivered-dose confirmation in humans. The current device operates as a research prototype and should be understood as a promising platform rather than an implementation-ready technology.

**On-patient immunization records.** Han and colleagues integrated intradermal mRNA delivery with record-keeping by embedding quantum-dot tags within the needle backing that remain in the skin after patch removal, producing a tamper-resistant record readable through a smartphone-linked near-infrared imaging device. The design addresses two simultaneous problems: thermolabile mRNA delivery and unreliable vaccination records in low-resource settings, where paper-based and centralized electronic systems depend on infrastructure and connectivity that cannot be assumed [5,229,230,231].

**Three-dimensionally printed latticed patches.** The microfluidic array patch delivers liquid or lyophilized self-amplifying RNA payloads from a single device with reduced application time, addressing the limitation of molded patches that required multiple units and 10 min application intervals to deliver clinically meaningful doses [117,157,232]. Digital traceability of each manufacturing parameter is compatible in principle with quality-by-design and continuous process verification, and per-batch geometric customization without new physical molds has been demonstrated at pharmacy-on-a-printer scale [233]. The published data derive from prototype-scale continuous liquid interface production under research conditions, and cGMP validation together with in-human measurement of delivered dose remains outstanding, thus the platform is presented here as promising rather than implementation-ready.

**Standardized applicators.** Kang and colleagues built a latch-driven applicator from three plastic components activated by thumb pressure on a trigger button, held within 20 mm length and 40 mm diameter, combining ease of use with inexpensive manufacture [234]. Spring-loaded single-use devices drive high-density patches into skin upon button release [235,236]. In a Vaxxas trial comparing trained-staff application with self-application at the upper deltoid, delivery performance was closely matched across arms [237].

**Theranostic and closed-loop devices.** Microneedles that sample interstitial fluid for antibodies, cytokines, and immune cell populations and transmit data wirelessly would enable real-time immune monitoring without blood draws; proof-of-concept work has recovered tissue-resident memory T cells at levels comparable to conventional sampling [238]. Hydrogel-forming arrays naturally draw and retain interstitial fluid, making the same swelling mechanism that drives release usable for biomarker capture [239]. Sensing needles acquire biosignals that integrated electronic modules condition, digitize, and relay wirelessly to smartphones, where algorithmic processing supports analysis, medication guidance, longitudinal records, and remote consultation [22,240,241]. A CRISPR-Cas9 wearable patch for cell-free DNA monitoring integrates nucleic acid extraction by reverse iontophoresis, CRISPR-based detection, and a graphene bioelectronic transducer within a single device [242]. Gene editing at the NLRP3 locus to silence the inflammasome driving pathological IL-1β and IL-18 secretion in psoriasis and atopic dermatitis offers potential long-term remission from a single application [243], although off-target editing risk in keratinocytes and skin-resident immune cells must be fully characterized before clinical translation.

## 10. Regulatory Science and Clinical Translation

Despite almost twenty years of preclinical investigation, few systems have reached clinical evaluation [158]. Sanofi Pasteur’s Fluzone remains the sole FDA-approved microneedle-delivered vaccine, a quadrivalent influenza product administered through a microinjection system rather than an array patch [244]. Vaxxas, Vaxess Technologies, and Micron Biomedical have emerged as the principal industrial actors, and the pace of clinical and manufacturing progress suggests that a first market-licensed array patch product may appear within five years [158].

The gap between preclinical promise and clinical reality reflects three barrier categories: scientific, where formulation, dose, and geometry fail to translate from murine to human skin; regulatory, where harmonized guidance for combination products does not exist; and commercial, where manufacturing scale-up and cost competitiveness remain unverified. The single Phase IIa negative outcome confirms that human clinical iteration remains necessary for design optimization [20,208].

Regulatory classification diverges across jurisdictions. The FDA classifies vaccine-loaded microneedles as combination products under 21 CFR 3.2, assigning a primary mode of action that directs review to the Center for Biologics Evaluation and Research with parallel Center for Devices and Radiological Health review of the device component [92]. Sponsors must therefore satisfy biological requirements covering sterility, potency, identity, purity, and stability alongside device requirements covering ISO 10993 biocompatibility, mechanical characterization, and user-factor studies for self-administration [53]. The Japanese Pharmaceuticals and Medical Devices Agency and Korean Food and Drug Administration follow a comparable framework, whereas the European Medicines Agency and the United Kingdom Medicines and Healthcare Products Regulatory Agency classify these products as integral medicinal products subject to conformity assessment [61,245]. WHO prequalification constitutes a third pathway, indispensable for low- and middle-income deployment, and the agency has issued a measles–rubella target product profile that informed both trial design and broader regulatory engagement [202].

FDA guidance issued in draft in 2017 and finalized in 2020 names needle length, array size, sharpness or penetration capability, and administration precision as quality attributes, yet targets cosmetic applications and remains incomplete for vaccine-loaded products [92]. A multi-stakeholder consensus proposed by Dul and colleagues covers biological attributes such as antigen identity and potency confirmed through validated immunoassays; chemical attributes including polymer composition, residual solvent, and degradation products; microbiological attributes including bioburden, sterility, and endotoxin; and physical attributes including geometry, dose uniformity within and across patches, mechanical integrity, dissolution kinetics, and packaging integrity [61,245]. No microneedle-specific Good Manufacturing Practice yet exists, and conventional injectable frameworks do not address mechanical integrity, coating uniformity, in-skin dissolution, or cargo stability within solid matrices [246].

Sterility assurance remains the most critical unresolved attribute, and the standard of proof is stringent. A parenteral product that breaches the stratum corneum must satisfy a sterility assurance level of 10^−6^, meaning a probability of no more than one non-sterile unit per million manufactured, per ISO 11137-2 [247]. Since sterile filtration cannot be applied to a solid-dose device, the choice narrows to terminal treatment of the finished patch or aseptic processing throughout, and every principal terminal method damages thermolabile cargo, the polymer matrix, or both.

Radiation chemistry acts on cargo and matrix through separate mechanisms. Gamma irradiation at 25 kGy, the reference dose adopted under the ISO 11137-2 VDmax25 method for SAL 10^−6^, generates hydroxyl radicals and hydrated electrons that cleave the mRNA phosphodiester backbone through direct strand scission and free-radical addition to the ribose ring, with measurable loss of translatable transcript at doses as low as 5–10 kGy in isolated systems [187,248]. On lipid nanoparticles, the same chemistry drives peroxidation of the ionizable amino lipid and the DSPC or DOPE helper phospholipid, altering bilayer packing, increasing mean hydrodynamic diameter, and reducing mRNA encapsulation efficiency, with those changes correlating with loss of in vitro translation and in vivo protein expression [187,248]. Electron-beam radiation at 10–25 kGy operates through the same free-radical chemistry but deposits energy over milliseconds rather than hours, limiting thermal accumulation and, for some antigens, preserving activity. Monovalent inactivated influenza hemagglutinin in a carboxymethylcellulose dissolving matrix retained immunogenicity indistinguishable from the non-irradiated control at 10 and 20 kGy [119], whereas model protein cargoes including ovalbumin in polyvinylpyrrolidone-based dissolving arrays lost antigenic integrity at 25 kGy despite matrix preservation [138]. Sterilization compatibility is therefore antigen-specific rather than platform-specific and must be re-validated for each new cargo.

Polymer matrices respond in class-specific ways. Aliphatic polyesters such as PLGA and PLA undergo predominantly chain scission at 25 kGy, with number-average molecular weight decreasing by 15–40% and consequent acceleration of hydrolytic degradation and payload release [249]. Polyvinylpyrrolidone and polyvinyl alcohol undergo simultaneous scission and radical–radical recombination, with the balance shifting toward cross-linking in the dry solid state and toward scission in aqueous solution; hyaluronic acid undergoes glycosidic-bond cleavage, with viscosity and mechanical strength decreasing sharply above 15 kGy [249]. Moist processing worsens each of these effects, while low-temperature irradiation (–70 °C to –80 °C) and packaging with radical scavengers such as ascorbate, mannitol, or methionine reduce them, defining an operating range narrower than the one used for conventional device sterilization [138,249]. Dry heat at 160 °C for 2 h and moist autoclave cycles at 121 °C for 15 min denature protein antigens and disrupt lipid nanoparticle organization within minutes of exposure, and remain compatible only with metallic solid and hollow needles loaded post-sterilization [53,70]. Thermal reflow polishing of laser-machined or micromolded needle tips, sometimes proposed to smooth surface irregularities, requires temperatures of 150–200 °C for PLA and PLGA and is likewise incompatible with cargo-loaded patches, thus the operation must be completed on the empty scaffold before antigen deposition [138].

Aseptic processing under isolator containment is therefore the only viable route for thermolabile microneedle patches, and four engineering requirements remain incompletely addressed. Contained isolators operating at ISO 5 (Grade A) provide the barrier of choice, superseding open cleanrooms because operator-borne particulate is the dominant contamination source in micron-scale filling. First, glovebox-mounted precision dispensers must place microliter formulations into arrays whose individual needle cavities hold only a few nanoliters, while maintaining unidirectional laminar airflow at 0.36–0.54 m/s, since turbulence around the array disrupts droplet placement and can carry particulates into open cavities [250]. Second, vacuum-based mold filling, which the Vander Straeten printer uses to eliminate air pockets in tip-loaded formulations, requires isolator-compatible low-pressure enclosures and vacuum lines whose HEPA-filtered inlet air remains at Grade A quality upon pressure release; the transient depressurization–repressurization cycle is a recognized hazard for microbial ingress and requires validated leak-tight subassemblies [117,250]. Third, controlled drying under humidity and temperature ramps of several hours must proceed within the same barrier without operator intervention, and integrating aseptic drying chambers with a vapor hydrogen peroxide decontamination cycle is constrained because residual VHP oxidizes both mRNA and ionizable lipids at concentrations above roughly 1 ppm, requiring extended aeration and confirmatory residual assays before every batch [250]. Fourth, patch removal from molds and transfer to primary packaging must remain fully isolated, since manual intervention at this stage negates all upstream control, and although robotic patch retrieval, in-isolator lidding, and continuous environmental monitoring have all been demonstrated individually for other combination products, none has yet been integrated end-to-end for a microneedle line [117,250]. Validation of the printer, the microfluidic patch, and the CLIP-based platforms under these constraints has not yet been reported [117,159], and the resulting engineering program is likely to define the critical path to first commercial licensure as decisively as any remaining immunological question.

Dose and content uniformity present parallel difficulties. USP General Chapter <905> Uniformity of Dosage Units frames the requirement through an acceptance-value calculation on 10 units at Level 1 and on up to 30 units at Level 2, with individual unit content expected to fall within 85–115% of label claim for most products [251]. Translating this framework to microneedle patches raises interpretive questions that no harmonized guidance has yet answered. If the finished patch is treated as a single dosage unit, the pharmacopoeial thresholds apply directly, but the within-patch variability across individual needles becomes invisible to the acceptance calculation. If each needle is treated as an independent subunit, hundreds of measurements per patch become necessary, and the routine assays used for solid oral or parenteral products, principally high-performance liquid chromatography and enzyme-linked immunosorbent assay, lack the sensitivity required at the sub-microgram scale of a single needle [252]. A microneedle-specific interpretation of <905> that recognizes both the total dose per patch and the distribution across the array is required, and the Dul multi-stakeholder consensus discussed above provides a starting point for that specification [61,245].

Two-step casting, the dominant micromolding method for clinically staged dissolving patches, introduces several routes to non-uniformity. In the first step, a concentrated antigen solution is dispensed onto the mold surface, drawn into the needle cavities under vacuum or centrifugation, and dried; in the second, a backing polymer is cast over the primary layer. Antigen distribution is governed by the volume dispensed, cavity geometry, ink rheology, drying rate, and the interfacial diffusion that occurs when the second-cast solution rewets the primary layer. Reported coefficients of variation for total dose per patch typically fall between 5% and 15% within a batch and can widen to 10–20% between batches under academic conditions, values that would fail a strict <905> acceptance calculation without process optimization [117,138]. Vacuum-based mold filling, inks engineered to gel rapidly after dispensing, and controlled staged drying reduce these coefficients, while polydimethylsiloxane mold shrinkage, absorption of small hydrophobic molecules by the elastomer, and lot-to-lot variability in polymer molecular weight and moisture uptake set an irreducible floor on achievable uniformity for elastomeric molds under Good Manufacturing Practice conditions [138].

Distributed 3D printing and inkjet fabrication address several of these problems at the source and reframe the between-batch question as a between-site question. The Vander Straeten microneedle vaccine printer achieved a standard deviation of 1.6 µg per patch across a hundred-mold tray for DNA loading with no positional trend, and encapsulated mRNA loading of approximately 1.0 µg per patch with low batch-to-batch variation, values well within a plausible <905>-compatible specification for a sub-microgram product [117]. The microfluidic microarray patch produced by continuous liquid interface production carries lyophilized cargo in defined reservoirs and removes the tip-loading step altogether [159]. Inkjet printing, which deposits picoliter-to-nanoliter droplets under piezoelectric or thermal actuation, can in principle achieve intra-patch coefficients of variation below 5% for coated arrays, subject to nozzle clogging, ink viscosity drift, and drying-induced coffee-ring artifacts that closed-loop droplet-volume feedback partly corrects [253]. Under the decentralized manufacturing model envisioned for a distributed vaccine-printer network, between-printer reproducibility becomes a separate critical quality attribute, requiring shared master files for ink formulation, mold geometry, and process parameters, together with harmonized batch-release testing and inter-site proficiency programs analogous to those operated for cell and gene therapy point-of-care manufacture.

In-line process analytical technology offers the most credible route to real-time content-uniformity assurance and moves quality verification from end-product acceptance sampling to build-in assurance. The FDA framework for process analytical technology, issued as guidance in 2004, promotes timely measurement of critical quality and performance attributes so that variability is understood and controlled rather than detected only at final release [254,255]. Near-infrared spectroscopy is the most industrially mature PAT tool for solid dosage forms, permits non-destructive bulk measurement within seconds, and is particularly well suited to residual moisture determination, a parameter directly relevant to lipid nanoparticle stability, with detection limits below 0.1% *w*/*w* when calibrated by partial least squares regression against Karl Fischer titration on representative reference batches [254,255]. Raman spectroscopic imaging complements near-infrared by providing chemical selectivity at spatial resolution of 1 to 2 µm, sufficient to map antigen and polymer distribution across individual needles and to detect segregation between tip and backing. Confocal Raman further permits depth profiling through the needle body, and successful pharmaceutical implementation of in-line Raman includes freeze-drying end-point detection and blend uniformity monitoring for oral solid dosage forms [256]. Machine vision paired with deep-learning geometric-conformance classifiers adds a low-cost real-time layer for morphology screening, catching malformed, incompletely tipped, or fractured needles before packaging. Integration of these tools into the aseptic isolators described earlier remains an open engineering task, since near-infrared and Raman probes must maintain optical alignment through glovebox windows or endoscope-mounted geometries without breaching the Grade A barrier, and chemometric models must be trained and revalidated whenever excipient composition, cargo concentration, or mold geometry changes. Delivery efficiency in the skin remains a further contributor to functional variability, since deposition depends on passive diffusion, the pattern of microchannels generated on insertion, and initial antigen concentration; comprehensive datasets combining PAT-derived content-uniformity data with insertion-force and dye-tracer readouts under a quality-by-design framework offer the most direct path toward a regulatory specification that captures both manufactured and delivered dose [158].

## 11. Manufacturing Scale-Up, Safety, and Global Health Implementation

### 11.1. Scale-Up and Cost of Goods

Current capabilities support Phase I and Phase II supply, but Phase III and commercial rollout require mass-production methods backed by substantial industry investment [158]. Even the most advanced automated printer and microfluidic patch platforms remain at prototype scale, have not been operated under current Good Manufacturing Practice, and have not documented delivered-dose accuracy in humans; each is more properly characterized as a promising manufacturing concept than as an implementation-ready system, and the transition to cGMP-compliant production is a discrete milestone rather than an incremental refinement [117,158,159]. Four challenges dominate: mold fabrication at industrial scale with consistent quality attribute compliance; aseptic filling of liquid nucleic acid formulations into micron-scale cavities without contamination or cargo damage; controlled drying that preserves lipid nanoparticle integrity while achieving the low residual moisture required for ambient stability; and packaging that maintains moisture barrier properties across a multi-month shelf life [53]. Aseptic isolators, though mature in conventional biopharmaceutical manufacture, require modification to accommodate precision dispensing, vacuum-based mold filling, controlled drying, and patch removal, and integrating a decentralized printer with a cleanroom-compatible isolator remains an unresolved engineering problem [53,117].

Life-cycle modeling indicates that removing cold-chain requirements could reduce total delivery cost in low- and middle-income countries by 50–80%, potentially more than offsetting any manufacturing premium relative to vial-based injection. Added upfront expense originates from stabilizing excipients, novel production technologies, and training for clinicians or self-administering patients [198]. Cost-effectiveness depends on dose size and administration frequency: extended shelf life offsets the expense of frequently administered vaccines, whereas rarely used products may not justify the investment [92]. The modeling literature converges on a conditional finding, namely that microneedles are significantly more cost-effective than needle and syringe specifically in childhood immunization programs with substantial coverage gaps or cold-chain losses rather than uniformly across all contexts [257,258]. This qualification tempers any unconditional economic claim, and the economic case is strongest in the low-resource settings where empirical cost data remain sparsest [36].

Single-dose presentation removes the multi-dose vial wastage that consumes a substantial proportion of total vaccine loss in low- and middle-income countries, and constitutes a target product profile requirement [202]. Packaging requires attention across storage, shipping, and use, with aluminum and plastic laminates providing moisture and environmental protection [92,259].

Public–private coordination has been required for successful microneedle development. The Vaccine Innovation Prioritization Strategy Alliance, comprising the Gavi Secretariat, the World Health Organization, the Bill & Melinda Gates Foundation, UNICEF, and PATH, selected three innovations for prioritized development in May 2020, of which microarray patches and thermostable qualified vaccines accounted for two [260]. A five-year action plan committed to defining the requirements needed to accelerate patch-based vaccines for low- and middle-income countries [260]. Communities historically underserved by immunization services, including those affected by poverty, Indigenous groups, people with disabilities, and residents of rural or remote areas, stand to gain meaningfully, as do older adults and immunocompromised patients through prioritized pathways and feasible mass campaigns [36,261,262].

### 11.2. Safety, Tolerability, and Acceptability

Local reactogenicity is the most common adverse event and is mechanistically expected as a correlate of efficacious intradermal delivery rather than a safety signal. Erythema, induration, pruritus, and discoloration are typically mild and self-limited and resolve within days to weeks. Barrier integrity recovers within 48 h and erythema resolves over roughly seven days, with long-term function intact even after high-density application [44,45,263]. Fourteen studies assessing safety within the Berger systematic review recorded no serious treatment-related adverse events [36], and no scarring has been reported [55], in contrast to the scarring risk of repeated hypodermic injection.

The Adigweme trial characterized pediatric reactogenicity in greater detail than any prior study. Mild induration affected 77% of toddlers and 65% of infants receiving the vaccine patch versus 15% and 10% of placebo-patch recipients, confirming an antigen-driven rather than purely mechanical etiology; all events were Grade 1 and none required treatment [37]. Systemic solicited events were comparable between groups, and fever occurred less often in patch-treated toddlers (8%) than in subcutaneous controls (18%) [37]. No related severe or serious adverse events and no clinically significant laboratory changes occurred across all three cohorts.

Application-site hyperpigmentation was the most frequent related unsolicited event, affecting 48% of toddlers and 83% of infants receiving the vaccine patch and 20% and 53% of placebo-patch recipients, confirming the polymer matrix rather than viral antigen as the principal driver [37]. Post-inflammatory hyperpigmentation occurs more frequently and more prominently in darkly pigmented skin because constitutive melanogenic activity is greater and melanocytes respond more strongly to inflammatory mediators. More than half of these reactions cleared by day 42 and nearly all by day 180, although 5% of infants retained discoloration at that point [37]. These outcomes are properly classified as cosmetic and acceptability considerations rather than safety liabilities, yet in populations where pigmentation changes carry social significance the impact must be addressed transparently during informed consent and through post-marketing surveillance, with excipient composition and applicator pressure optimization as development priorities [37,202].

Patient-reported outcomes consistently favor microneedle patches. Preference exceeded 70% at 28 days in the Phase I influenza trial [33,34], and most parents in the Gambian trial preferred patch delivery over needle and syringe. Realistic deployment is likely to proceed in phases, beginning with trained medical staff, moving to trained community workers, and eventually reaching user-applied administration [158]. Australian clinicians accepted self-administration within clinics but remained wary of unsupervised home use, citing anaphylaxis risk and documentation gaps [36,232,235,264]; tamper-resistant on-patient records offer one practical remedy. Selection among these modes should follow the antigen safety profile, target population, healthcare infrastructure, acute-adverse-event risk, and surveillance requirements. The measles–rubella patch is designed for administration by individuals who are not healthcare professionals [32,37], substantially expanding the vaccination workforce and potentially reaching zero-dose children inaccessible to conventional services.

## 12. Challenges and Future Perspectives

**Figure 4:**
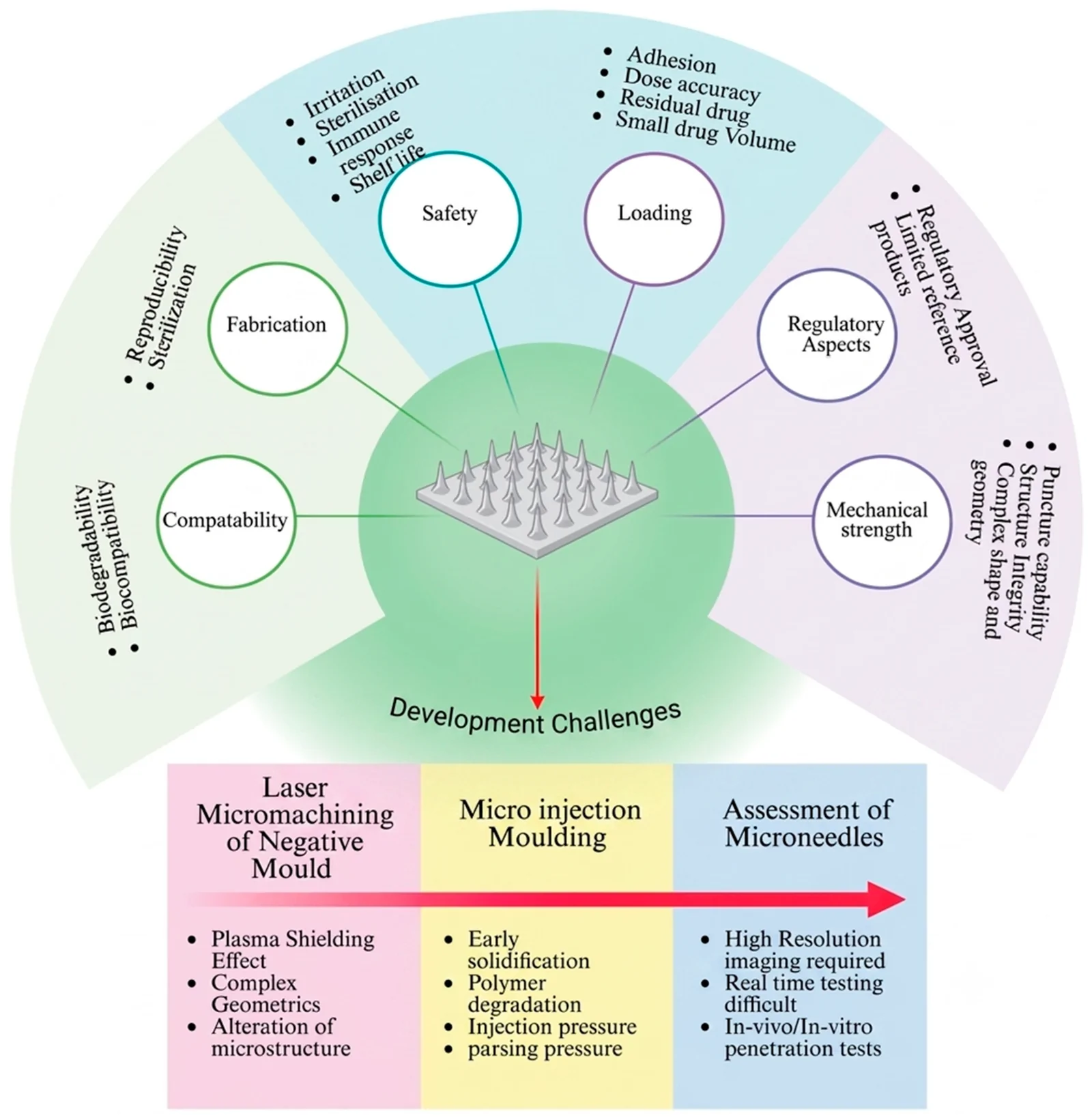
Interdependent technical, regulatory, and translational challenges constraining microneedle clinical deployment. Six connected domains define the microneedle development: biocompatibility and biodegradability of matrix materials; reproducibility and sterilization of the fabrication process; safety attributes including irritation, immune response, and shelf life; cargo loading constraints including adhesion, dose accuracy, residual drug, and small volume per needle; regulatory aspects including the limited number of reference products available for comparative submission; and mechanical strength relating to puncture capability, structural integrity, and complex geometric design. The lower panel illustrates fabrication-specific challenges in laser micromachining of negative molds, micro-injection molding, and microneedle assessment. Images reprinted with permission from [84].

Six interlocking obstacles continue to constrain deployment, and progress in each frequently depends on advances in the others. Figure 4 illustrates these interdependent domains.

**Insertion depth and dose variability.** Skin thickness and mechanical properties differ both between individuals and across body sites, thus penetration depth and release vary even under identical pressing force and speed [19,22]. The painless effective window of approximately 100–1100 µm is considerably narrower than the range of human skin thickness variability, implying that one-size-fits-all geometries cannot achieve optimal dosing across diverse populations [55,151]. Standardized applicators constrain this variability within an acceptable operating window [77,265,266], and quality-by-design mapping of insertion force, velocity, and depth against immunological readouts will be required to define regulatory-grade specifications.

**Payload capacity.** Patches deliver sub-milligram quantities, with larger formats reaching roughly 10 mg [267,268] and solid-state coating or molding typically yielding under 10 µg per needle [77]. Expansion strategies include greater patch area, higher needle density, longer needles within the painless window, and reservoir-based hollow or hydrogel configurations. Faceted printed geometries offering 36% higher loading [153] and microfluidic reservoir designs [157] represent the principal routes forward.

**Nucleic acid stability.** Drying lipid nanoparticles within a solid matrix requires simultaneous preservation of chemical integrity and colloidal stability [187], and the restricted polymer volume inside a needle aggravates the difficulty of suppressing aggregation during drying [117]. The polyvinylpyrrolidone–polyvinyl-alcohol matrix achieving six-month ambient stability represents the field’s most significant advance [117], yet the full set of formulation parameters affecting integrity through fabrication, storage, and in-skin dissolution remains incompletely characterized. Standardized analytical methods including capillary electrophoresis, cryo-electron microscopy, and transfection assays will be essential for regulatory submissions [55].

**Immune response variability and evidentiary interpretation.** The Berger systematic review reported a standardized mean difference of 10.80 (95% CI 3.51–18.08) favoring patch-delivered influenza hemagglutination inhibition titers over intramuscular injection, but with extreme heterogeneity (I^2^ = 99%) driven substantially by a single outlier study [36,62]. An effect of this magnitude reflects clinical and methodological diversity rather than uniform biological superiority. Patch-delivered influenza antigen achieves immunogenicity non-inferior to intramuscular injection, with dose-sparing rather than dose-equivalent superiority as the principal value proposition. This distinction matters for product strategy, as overstating immunogenic superiority could misdirect development away from the contexts where the platform genuinely excels.

**Long-term polymer safety.** Concerns extend beyond acute reactogenicity to inflammation from repeated application, systemic exposure to degradation products, and immunological responses to novel matrices [22]. Well-characterized polymers carry favorable profiles, but the cumulative effect of repeated intradermal exposure across years of vaccination schedules has not been characterized. Stimuli-responsive polymers, printing photopolymer resins, and emerging biopolymers require ongoing surveillance for delayed reactions, sensitization, and effects on barrier function.

The advancement beyond these obstacles is shaped by convergence with adjacent technologies. Machine learning applied to the high-dimensional geometric, material, and formulation parameter space can identify optimal combinations from the large structure-activity datasets currently available [151,269,270], and coupling predictive models to automated printers would close the loop between computational design and physical production, supporting digital customization per batch without new molds [117,233]. Four-dimensional printing extends this capability by incorporating time-dependent shape change and adaptive geometry, permitting patches whose dissolution kinetics adjust to skin temperature or hydration. Wearable sensor-integrated devices will connect real-time biomarker capture with algorithmic decision support, generating longitudinal records of vaccine-induced humoral and cellular responses that support personalized booster scheduling and rapid identification of poor responders [22,238,240]. Prime–pull strategies use an intradermal prime to seed systemic immunity that a subsequent mucosal boost refines, potentially generating serum antibody, tissue-resident memory T cells, and mucosal IgA from a single platform. Pan-pathogen designs targeting conserved hemagglutinin stem regions, conserved spike epitopes, or conserved flavivirus structural elements would address the strain-specific reformulation requirement, and the demonstrated capacity for simultaneous multi-cargo tip-loading [117] and multi-stage stimuli-responsive release [271] supplies the delivery capability. The remaining obstacle is formulation chemistry capable of stabilizing multiple antigens simultaneously within a solid matrix. Table 8 provides a structured research roadmap with falsifiable five- and ten-year milestones.

**Figure 5:**
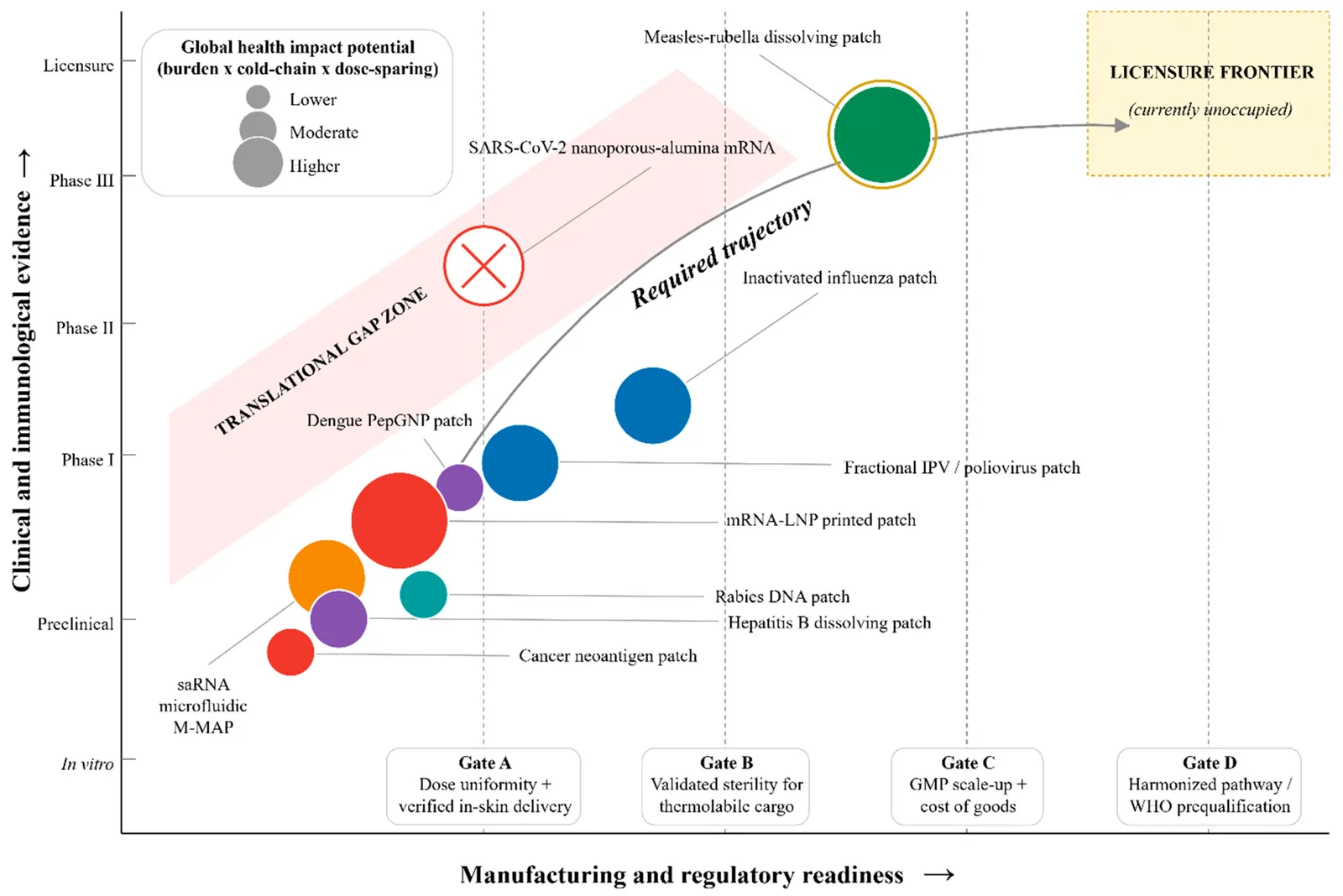
The translational gap landscape of microneedle vaccines. Each node positions a vaccine target and platform combination according to clinical and immunological evidence (vertical axis, from in vitro proof-of-concept through licensure) and manufacturing and regulatory readiness (horizontal axis, gated by four sequential requirements: (Gate A) dose uniformity with verified in-skin delivery, (Gate B) validated sterility assurance for thermolabile cargo, (Gate C) Good Manufacturing Practice scale-up with acceptable cost of goods, and (Gate D) harmonized regulatory approval or World Health Organization prequalification). Node area encodes global health impact potential, informed by disease burden, cold-chain dependence, and, where demonstrated, dose-sparing; node color denotes cargo modality (green, live-attenuated; blue, inactivated; red, mRNA; orange, saRNA; purple, subunit; teal, DNA). The shaded band marks the translational gap zone, within which immunological validation is strong yet manufacturing and regulatory readiness lags. The measles–rubella dissolving patch alone approaches the licensure frontier, which remains unoccupied, and therefore defines the trajectory that subsequent programs must follow. The nanoporous alumina SARS-CoV-2 patch, marked as an unmet endpoint, demonstrates that advanced clinical stage does not compensate for unoptimized needle geometry and payload release.

Figure 5 positions the field’s principal programs within a shared space defined by clinical evidence and manufacturing readiness, exposing a systematic pattern that individual trial reports cannot reveal. Nearly every target and platform combination lies toward the left of the plane, where preclinical or human data establish immunogenicity, dose-sparing, or thermostability, yet manufacturing and regulatory readiness remains immature. Programs with the strongest human evidence populate the translational gap zone in the upper left, while earlier-stage candidates cluster in the lower left, and in neither group has readiness kept pace with immunological promise. This distribution substantiates the central conclusion of the present review, namely that the decisive obstacles have shifted from immunology to industrialization. The measles–rubella dissolving patch alone approaches the licensure frontier, having demonstrated pediatric immunogenicity and safety, secured an established World Health Organization target product profile, and advanced to Phase III with a defined path to prequalification, and therefore defines the template trajectory for subsequent programs. Decentralized mRNA and self-amplifying RNA patches carry impact potential rivaling the measles–rubella patch, yet are positioned farthest from the frontier, constrained primarily by sterility assurance for thermolabile cargo and by unvalidated Good Manufacturing Practice fabrication. The nanoporous alumina SARS-CoV-2 outcome occupies an instructive position, since clinical advancement to Phase IIa did not offset unoptimized geometry and payload release, confirming that readiness gates cannot be bypassed through immunological rationale alone. The empty upper-right region defines the collective objective for the coming decade. Prioritizing investment along the horizontal readiness axis, rather than accumulating further immunological proof, offers the most direct path toward licensure and equitable deployment.

## 13. Conclusions

Microneedle-based vaccination has advanced from laboratory concept to clinically validated technology supported by credible regulatory routes, working manufacturing prototypes, and coordinated public–private investment. The biological rationale relies on the immunological density of skin, where resident Langerhans cells and dermal dendritic cells capture antigen and traffic to draining lymph nodes far more efficiently than muscle permits, yielding germinal-center responses and, in human trials, six-fold dose-sparing for inactivated influenza and four-fold for inactivated poliovirus; comparable observations for nucleic-acid SARS-CoV-2 candidates remain confined to preclinical species. Solid-state formulation converts this immunological advantage into a logistical one, preserving antigen potency at ambient and high temperatures for periods reaching two years and removing the cold-chain dependence that constrains conventional immunization programs. The product translation now depends less on immunology than on industrialization. Sterility assurance for thermolabile cargo, dose uniformity, nucleic-acid stability within solid matrices, and harmonization across divergent regulatory frameworks remain the principal obstacles, alongside a clinical pipeline still narrowly concentrated on a few antigens. Resolving these issues will require sustained investment matched to the settings where the technology matters most, since cold-chain elimination and single-dose presentation yield the greatest returns where vaccine-preventable mortality is highest. The evidence assembled in this review indicates that microneedle vaccination is no longer speculative, and that the coming decade will determine whether accumulated promise becomes equitable delivery.

## Figures and Tables

**Table 1:** Comparative deposition, antigen retention, and immunological outcomes across delivery routes and graded skin depths.

Delivery Route (Approximate Depth)	Principal Target Compartment	Predominant APC Engaged	Reported Antigen Retention at Deposition Site	Reported Outcome for Lymphatic Delivery and Immune Response Arm	References
Transcutaneous (stratum corneum, <30 µm)	Epidermis via follicular route	Langerhans cells	Not systematically reported	CD8^+^ effector responses with mucosal IgA; systemic IgG absent	[59]
Intradermal microneedle, shallow (~100–300 µm)	Viable epidermis, dermo-epidermal junction	Langerhans cells	Sustained (≥3 days for particulate influenza antigen in mice)	Prolonged antigen availability; DC maturation and migration to draining nodes at 24 h	[57]
Intradermal microneedle, intermediate (~300–700 µm)	Papillary dermis (dermal DC- and lymphatic-capillary-rich)	Dermal dendritic cells	Sustained relative to IM (days)	Both humoral and cellular responses; efficient lymphatic drainage of small (~25 nm) particulates	[57,58,59]
Intradermal microneedle, deep (~700–1100 µm)	Upper reticular dermis (blood-vessel-rich)	Dermal DCs and macrophages	Not depth-stratified in primary literature	Progressive shift toward vascular clearance; response character not yet resolved from ID at shallower depth	[49,60]
Subcutaneous (4–15 mm)	Adipose tissue	Interstitial DCs and macrophages	Rapid clearance relative to intradermal	Antigen-specific IgG; limited antigen-specific CD8^+^ T cell induction	[59,60]
Intramuscular (20–40 mm)	Skeletal muscle	Resident tissue macrophages	Rapid clearance (highly vascularized bed)	Reference route; strong humoral response; muscle lacks resident DC network of skin	[54,60]

**Table 2:** Dose-sparing effects of microneedle-delivered vaccines relative to intramuscular or subcutaneous comparators.

Vaccine and Platform	Comparator Route	Dose Reduction	Study Level (Species/Phase)	Primary Endpoint Outcome	Source
Inactivated influenza (HD-MAP, Vaxxas)	Intramuscular, 15 µg HA	6-fold (2.5 µg HA)	Clinical, Phase I, healthy adults	Hemagglutination inhibition titer non-inferior to intramuscular	[62]
Influenza (coated microneedle)	Intramuscular standard	3-fold	Preclinical, mice	Higher hemagglutination inhibition and memory B cell frequency	[64]
SARS-CoV-2 DNA subunit (electroporation-enabled solid MN)	Intramuscular	10-fold	Preclinical, rats	Equivalent neutralizing antibody titers	[65]
Inactivated poliovirus (fractional intradermal, MicronJet600)	Conventional intramuscular	4-fold	Clinical, multiple trials; WHO SAGE-endorsed	Equivalent seroconversion rates	[63]
Tuberculosis Ag85B DNA	Intramuscular	Comparable at 4.2 µg; superior at 12.6 µg	Preclinical, mice	Antibody titer and antigen-specific IFN-γ	[66]

**Table 3:** Comparative summary of the principal microneedle classes for vaccine delivery.

Class	Mechanism	Typical Materials	Dose Capacity	Principal Advantages	Principal Limitations	Representative Vaccine Application
Solid (poke-and-patch)	Two-step: penetrate skin, then apply topical formulation	Silicon, stainless steel, titanium, PLA	Low (diffusion-limited)	High mechanical strength; simple arrays	Two-step use; rapid channel closure; residual surface drug; non-degradable waste	DNA vaccine with electroporation [65]
Coated (coat-and-poke)	Surface film dissolves upon insertion	Metal or polymer base with coating layer	Low to moderate (<10 µg per needle)	Single-step delivery; rapid release; precise dosing	Limited loading; coating shear loss; humidity sensitivity	Phase I influenza dose-sparing trial [62]
Hollow (poke-and-flow)	Pressure-driven flow through a lumen	Silicon, metal, ceramic, glass, polymer	High (reservoir-limited)	Accepts liquid formulations; large volumes	Reduced strength; clogging and leakage; pain reports	MicronJet600 influenza, rabies, BCG trials [82]
Dissolving (poke-and-release)	Polymer matrix dissolves in interstitial fluid	PVP, PVA, CMC, HA, PLGA, sugars	Moderate (tip-loaded micrograms)	Single-step; no sharps; cold-chain reduction; self-application	Tip volume limits dose; humidity sensitivity; scale-up	Phase I influenza [34]; Phase I/II measles–rubella [37]
Hydrogel-forming (poke-and-swell)	Swelling-mediated diffusion from a reservoir	Gantrez AN-139, GelMA, methacrylated HA	High (reservoir-based)	Larger reservoirs; clean removal; ISF sampling	Inconsistent swelling; leakage; mechanical weakness	Preclinical ovalbumin model [88]
Hybrid and bioinspired	Porous, rapidly separating, cryogenic, core–shell, stimuli-responsive	Multiple combinations	Variable	Specialized functions: thermostability, on-demand release, live-cell delivery	Manufacturing complexity; multistep processes	Cryo-MN COVID-19 mRNA [91]; core–shell S1-RBD [93]

**Table 4:** Mechanical properties, advantages, limitations, and representative vaccine applications of the principal microneedle material classes.

Material Class	Representative Material	Tensile Strength/Young’s Modulus	Advantages	Limitations	Representative Vaccine Application
Silicon	Silicon	6900 MPa/130–188 GPa	High strength; mature microfabrication	Brittle; low loading; high cost; poor sustainability	Nanoporous npMNA, Phase IIa mRNA-1273 [20]
Metal	Stainless steel	580 MPa/193 GPa	Widely available; scalable; biocompatible	Corrosion; nickel allergy risk; non-degradable	Coated influenza microneedles [75]
Metal	Titanium	240–550 MPa/102–120 GPa	Biocompatible; low corrosion	Rigid; non-degradable; high cost	Porous titanium delivery [83]
Ceramic	Alumina (Al_2_O_3_)	260 MPa/380–410 GPa	Biocompatible	Brittle; non-degradable	npMNA SARS-CoV-2 mRNA-1273 [20]
Natural biopolymer	Hyaluronic acid	≈40 kPa (PEG-cross-linked)	Biodegradable; CD44 receptor engagement	Low strength; limited loading	Trivalent influenza HA [131]; Raphas products [132]
Natural biopolymer	Chitosan	Variable	Intrinsic adjuvant; antimicrobial; biodegradable	Multistep processing; poor solubility	Implantable influenza chitosan MN [107]
Natural biopolymer	Silk fibroin	Variable	β-sheet stabilization; low cost	Fractures easily as a base material	Polio (3 years, RT, 70% potency) [112]
Synthetic polymer	PLGA	Variable	Biodegradable; sustained release	High cost; hydrophobic	Ovalbumin with poly(I:C), hollow MN [114]
Synthetic polymer	PVP	Variable	Rapid dissolution; regulatory acceptance	Not biodegradable in humans	mRNA–LNP vaccine printer [117]
Synthetic polymer	PVA	Variable	Superior LNP stabilization; film-forming	Slow drying; hygroscopic	PVP–PVA blend for mRNA–LNP MN [117]
Synthetic polymer	CMC	Variable	Biocompatible; film-forming	Hygroscopic; substitution-dependent	Recombinant coronavirus dissolving MN [133]
Sugar matrix	Trehalose/sucrose	Temperature- and humidity-sensitive	Vitrification and water replacement	Humidity sensitive	DTP–HepB–Hib, 25 °C, 12 months [30]; influenza, 25 °C, 12 months [29]
Smart material	PNIPAM (thermoresponsive)	Variable	On-demand release near body temperature	Restricted to specific triggers	Insulin and anticancer release [134,135]
Smart material	Ultra-pH-responsive copolymer	Variable	Tumor-microenvironment targeting	Chiefly oncology applications	Cancer immunotherapy MN [136]

**Table 5:** Fabrication technologies for vaccine microneedles: resolution, cargo compatibility, and representative applications.

Technique	Resolution/Scale	Vaccine Compatibility	Advantages	Limitations	Representative Application
Micromolding (PDMS)	≈10 µm features; batch scale	All classes, with controlled drying	Simplicity; mature scale-up; tip-loading feasible	Mold dimensional stability; absorbs hydrophobic molecules	Phase I influenza [34]; MRV–MN patch, The Gambia [37]
Photolithography	Sub-100 µm; high throughput at scale	Post-fabrication coating only	Mature cleanroom workflows	Multistep; costly; ≈100 °C steps damage antigens	Silicon solid MN platforms [77,142]
Laser ablation and cutting	µm scale; high precision	Coating-based loading	Geometry customizable through CAD	Setup cost; thermal exposure	Stainless steel coated influenza MN [64]
Centrifugal and draw lithography	µm scale; pilot throughput	Compatible with biological cargo	Ambient temperature; commercial in Korea	Throughput limited at industrial scale	Raphas hyaluronic acid dissolving MN [132]
Stereolithography (SLA)	High resolution; smooth finish	Limited (water-insoluble photopolymer)	Mature commercial systems; CAD-driven	Photopolymer constraint; UV exposure	Insulin-coated SLA MN [146,149]
Digital light processing (DLP)	High resolution; faster than SLA	Limited (photopolymer)	Faster prints; CAD-driven	Material constraint; post-processing	Amoxicillin GelMA hydrogel MN [160]
Two-photon polymerization	≤100 nm features	Limited at scale	Highest resolution; complex geometries	Slow; costly; small build volume	Master molds and prototypes [161]
CLIP/iCLIP	≈100 µm features; high throughput	Compatible through tip-loading	Smooth surfaces; custom geometry; throughput	Resin selection; scalability	Faceted PEG MN [153]; saRNA M-MAP [157,159]
Inkjet printing	µm droplets; coating	Ambient; preserves cargo	Precise dose; no thermal exposure	Throughput; ink viscosity limits	Inkjet insulin coating [149,162]
Aerosol jet printing	Down to 10 µm; prototype scale	Proteins and peptides; risk to live-attenuated vaccines	Benign conditions; wide viscosity range	Poor reproducibility; complex parameters	Dissolvable PVP–trehalose MN [163,164]
Vaccine printer (MVP)	µm scale; 100 patches per 48 h	Optimized for mRNA–LNP through PVP–PVA	Automated; vacuum mold filling; thermostable output	Sterility under cGMP; throughput scale-up	Thermostable COVID-19 mRNA MN [117]
iCLIP M-MAP	µm scale; reservoir-integrated	Compatible with lyophilized LNP	Microfluidic delivery; spring-loaded applicator	Early-stage development	Lyophilized saRNA–LNP delivery [159]

**Table 7:** Principal clinical trials of microneedle vaccines.

Trial Identifier	Phase	Antigen or Indication	Platform	Sponsor	Population	Primary Endpoint Result	Source
NCT02438423	I	Inactivated influenza vaccine	Dissolving MN patch	Emory University; Georgia Tech	Healthy adults, 18–49 years	Safety and reactogenicity acceptable; immunogenicity non-inferior to intramuscular; 70% preferred patch	[34]
Rouphael 2021 substudy	I	Inactivated influenza vaccine	Dissolving MN patch	Emory University	Healthy adults	Higher neuraminidase inhibition titers and higher circulating Tfh frequency than intramuscular	[56]
Forster 2020	I	Inactivated influenza vaccine	HD-MAP	Vaxxas Pty Ltd.	Healthy adults	Six-fold dose-sparing at hemagglutination inhibition non-inferiority	[62]
NCT06125717	I	H1N1 influenza	MIMIX MAP	Vaxess Technologies	Healthy adults	Safety and immunogenicity across dose escalation (ongoing)	[200]
NCT04394689	I/II	Measles–rubella	Dissolving MN patch	Micron Biomedical; MRC Unit The Gambia; BMGF	Adults 18–40 y; toddlers 15–18 mo; infants 9–10 mo	93% measles and 100% rubella seroconversion in infants; no related serious adverse events	[37]
Prins proof-of-concept study	IIa	mRNA-1273 SARS-CoV-2	Nanoporous alumina npMNA	Leiden University Medical Center	Healthy adult volunteers	Spike S1 IgG booster response endpoint not met	[20]
NCT05315362	II	mRNA COVID-19 vaccine	Solid MN skin patch	Leiden University Medical Center	Adults	Immunogenicity and safety evaluation	[151]
NCT01813604	III	Polio (OPV, IPV, fractional IPV)	MicronJet600	Centers for Disease Control and Prevention	Various age groups	Immunogenicity comparison across formats	[151]
NCT01686503	II	Polio booster in HIV infection	MicronJet600	Eastern Virginia Medical School; NanoPass	HIV-positive adults	Intradermal versus intramuscular immunogenicity	[151]
NCT04064554	NA	BCG vaccination	MicronJet600	Yonsei University	Adults	Safety and immunogenicity versus conventional needle	[151]
NCT02621112	II/III	Hepatitis B in renal failure	MN with imiquimod adjuvant	The University of Hong Kong	Renal failure patients	Comparative immunogenicity	[151]
NCT03722472	I	Tuberculosis (ID93 + GLA-SE)	Thermostable formulation	—	Adults	Superior response versus non-stabilized form	[92,217]
PepGNP-Dengue	I	Dengue peptide–gold nanoparticle	Solid silicon MN	—	Adults	Immunogenicity reported; no microneedle-route comparator arm	[214]

**Table 8:** Structured five- and ten-year research roadmap for microneedle vaccines.

Domain	Five-Year Milestone	Ten-Year Milestone
Clinical pipeline	Phase III pivotal trial completed for the measles–rubella patch in pediatric low- and middle-income populations [37]	First WHO-prequalified microneedle vaccine licensed in two or more jurisdictions
Antigen diversification	Phase I/II trials for three or more WHO priority pathogens beyond influenza, Japanese encephalitis, and measles–rubella [36]	Pan-pathogen platform (universal influenza or pan-coronavirus) at Phase II
mRNA–LNP microneedles	Decentralized printer operating at GMP standard with regulatory submission [117]	Distributed mRNA manufacturing networks deployed across three or more LMIC regions
Self-amplifying RNA	First-in-human Phase I trial of an saRNA microneedle patch [159]	Licensed saRNA microneedle product for endemic infectious disease
Theranostic biosensing	Validated interstitial fluid biomarker assay deployed clinically [238,239]	Closed-loop immunization and monitoring system in routine care
Computational design	Validated machine-learning model for geometry and formulation optimization in product development [151,269,270]	Computationally designed platform showing two-fold or greater improvement in dose-sparing or immunogenicity
Cold-chain elimination	Three or more prequalified microneedle vaccines stable for 12 months or longer at 25 °C or above	Routine deployment of ambient-stable microneedle vaccines across tropical settings
Regulatory harmonization	Harmonized critical quality attribute guidance across FDA, EMA, PMDA, and WHO [61,245]	Mutual recognition agreements supporting global licensure
Manufacturing scale-up	Commercial-scale aseptic manufacturing line demonstrated for one or more products [246]	Five or more commercial facilities globally, including sites in LMICs
Sterility validation	Validated radiation-tolerant or aseptic process for mRNA–LNP microneedles [70,117]	Routine GMP sterility validation across multiple product classes
On-patient records	First clinical deployment of an on-body immunization record within an LMIC program [231]	Integration of microneedle immunization records with national digital health registries
Mucosal prime–pull	Phase I/II prime–pull vaccine for a respiratory pathogen with dual systemic and mucosal responses [215,216]	Licensed prime–pull platform with a validated mucosal correlate of protection
Cancer immunotherapy	First Phase I microneedle-delivered personalized neoantigen vaccine [219,272]	Licensed adjunctive microneedle cancer immunotherapy product
Pandemic preparedness	Operational decentralized printer network with rapid-response capability [117]	Demonstrated pandemic response within 100 days of pathogen identification

## Data Availability

No new data were created or analyzed in this study. Data sharing is not applicable to this article.
